# 8‐Oxoguanine Modified CircMTUS1 Drives PABPC1 Phase Separation to Promote Gastric Cancer Progression and Cisplatin Resistance via Autophagy

**DOI:** 10.1002/advs.76466

**Published:** 2026-07-09

**Authors:** Lei Peng, Lurong Li, Zhenghui Zhu, Chenyang Li, Huaiming Sang, Guoxin Zhang, Xuan Li, Yini Dang, Yuanyuan Li

**Affiliations:** ^1^ Department of Gastroenterology First Affiliated Hospital of Nanjing Medical University Nanjing China; ^2^ Department of Endocrinology Children's Hospital of Nanjing Medical University Nanjing China

**Keywords:** 8‐oxoguanine, autophagy, chemoresistance, circMTUS1, LLPS, PABPC1, stress granules

## Abstract

Gastric cancer (GC) is a major global health concern, as its prevention and treatment remain significant challenges. The 8‐oxoguanine (o8G) modification of circRNAs, alongside their capacity to orchestrate liquid‐liquid phase separation (LLPS) and autophagy, plays a pivotal role in driving tumor progression and determining therapeutic outcomes. Here, we identified the downregulation of circMTUS1 in GC and elucidated the upstream and downstream mechanisms governing its role in tumor progression and chemoresistance. We demonstrated that YBX1 induces o8G modification, which reduces circMTUS1 stability. Notably, circMTUS1 restoration markedly inhibits tumor growth both in vitro and in vivo. Mechanistically, circMTUS1 targeted PABPC1 through the LLPS to regulate stress granules (SG) formation and autophagy to inhibit GC progression and to affect the sensitivity to chemotherapy. Furthermore, the small‐molecule SG inhibitor ISRIB acts synergistically with circMTUS1 to significantly sensitize GC cells to cisplatin. Collectively, our data demonstrate that circMTUS1 suppresses GC progression through a pathway involving YBX1‐dependent o8G modification and PABPC1‐mediated regulation of SG and autophagy. Consequently, targeting circMTUS1 or its downstream SG may offer an effective strategy to enhance the chemosensitivity of gastric cancer.

## Abstract 2

circMTUS1 is downregulated in gastric cancer (GC), inhibiting progression and cisplatin resistance. Mechanistically, YBX1 acts as an o8G reader to reduce circMTUS1 stability, while circMTUS1 targets PABPC1 via liquid‐liquid phase separation to disrupt stress granule formation and cytoprotective autophagy. Combining circMTUS1 restoration or stress granule inhibition with cisplatin offers a promising therapeutic strategy for GC.

**Abbreviations**
3‐MA: 3‐methyladenine
5‐FU: 5‐fluorouracil
ActD: actinomycin D
CCK‐8: Cell Counting Kit‐8
circRNA: circular RNA
DDP): cisplatin
DOX: doxorubicin
EdU: 5‐Ethynyl‐2′‐deoxyuridine
FISH: Fluorescence in situ hybridization
GC: gastric cancer
H_2_O_2_
: hydrogen peroxide
IF: immunofluorescence
IHC: Immunohistochemistry
LLPS: liquid‐liquid phase separation
MMC: mitomycin C
MTUS1: microtubule associated scaffold protein 1
NAC: N‐acetylcysteine
NC: negative control
o8G: 8‐oxoguanine
PABPC1: poly(A) binding protein cytoplasmic 1
RAPA: rapamycin
RIP: RNA immunoprecipitation
ROS: reactive oxygen species
RT‐qPCR: real‐time quantitative polymerase chain reaction
SG: stress granules
siRNA: small interfering RNA
YBX1: Y‐box binding protein 1

## 1 Background

Gastric cancer (GC) is a growing public health concern worldwide especially in Asian countries. It ranks fifth as the most prevalent cancer and is a cause of cancer‐related deaths globally [1, 2]. Over the past few decades, significant advancements have been made in the understanding and treatment strategies for patients with GC. Notably, a spectrum of clinical therapeutic interventions has been extensively utilized in the clinical management of GC. These therapeutic modalities have conferred substantial clinical benefits to patients while maintaining adverse effects within a clinically tolerable range. Nevertheless, the prevention and clinical management of GC remain confronted with considerable challenges that warrant further addressing [3]. Therefore, it is of great significance to explore the mechanism of GC occurrence and chemotherapy resistance in order to achieve early diagnosis and treatment, improve the prognosis of advanced patients, and improve the survival rate [4].

Circular RNAs (circRNAs) represent a category of RNAs generated via the process of back‐splicing, and was characterized by a covalently closed circle that lacks both a 5’ cap and a 3’ poly(A) tail [5]. These molecules exert their biological functions through interactions with intracellular functional macromolecules, such as RNA, proteins, and DNA [6]. Owing to their distinct expression patterns, robust stability, and specific localization in different tissues, circRNAs have garnered substantial interest in the fields of cancer diagnosis and therapeutic development [7]. For instance, circCAPRIN1 was found to directly bind to STAT2, thereby activating the transcriptional activity of ACC1. This process was further illustrated to regulate lipid metabolism and facilitate colorectal cancer progression [8]. Nevertheless, the role of circRNAs in protein binding during GC progression remains to be further investigated.

During these years, research into circRNAs’ roles in oncogenesis and tumor progression has advanced. Nevertheless, recent investigations have primarily centered on their modulation of downstream signaling pathways, and the upstream molecular mechanisms governing circRNAs have not been thoroughly clarified [9, 10]. Advances in RNA modification research have furnished new perspectives on how circRNAs expression change and explore circRNAs’ functions in malignant tumors [11, 12]. Specifically, 8‐oxo‐7,8‐dihydroguanosine (o8G) modification regulates RNA‒RNA interactions in a “redox‐dependent” way [13]. Notably, o8G modifications have been detected in circRNAs [14, 15] and microRNAs (miRNAs, e.g., miR‐124, let‐7, miR‐1), to impact RNA stability, specific targeting properties, and modulate gene expressions [16]. However, o8G modification of circRNAs in gastric cancer (GC) has not been reported.

Liquid–liquid phase separation (LLPS) is a physicochemical and thermodynamic process where biomacromolecules, such as proteins or nucleic acids, spontaneously separate into dense and dilute phases in solution, thereby reaching the minimum free energy state. Mounting evidence suggests that LLPS underpins the formation of membrane‐less organelles. These include nucleoli, paraspeckles, Cajal bodies, and promyelocytic leukemia bodies in the nucleus, along with stress granules (SG) and processing bodies in the cytoplasm. When driven by the cumulative effects of proteins or nucleic acids, LLPS promotes the formation of liquid‐like membrane‐less aggregate compartments. These compartments help organize and partition cellular functions and are involved in a wide range of biological processes [17]. Impairment of this process is associated with a range of diseases, including cancer [18]. Meanwhile, recent studies have shown that ncRNAs can modulate tumor development by regulating the phase separation of RBPs [19].

In this study, we found that circMTUS1 was downregulated in GC and correlated with GC progression. We also characterized YBX1 as a reader protein that bound to o8G‐modified circMTUS1, an interaction that destabilized the transcript and suppressed its abundance. Our data indicated that cytoplasmic circMTUS1 act as a stabilizer for PABPC1 by preventing its ubiquitination. The accumulation of PABPC1 not only promoted phase separation and the assembly of SG but also modulated autophagy, ultimately compromising the therapeutic efficacy of cisplatin in GC. Taken together, our findings define the functional role of circMTUS1 in GC progression and offer a potential foundation for developing novel GC therapeutic strategies.

## 2 Method

### 2.1 Clinical Specimens

For this study, we retrieved microarray data from the Gene Expression Omnibus (GEO) database, including GSE100170, GSE184882, GSE152309, and GSE248612. All data were analyzed and processed using R software. Regarding clinical samples, we collected tumor tissue and its paired normal tissues from 76 GC patients surgically treated at First Affiliated Hospital of Nanjing Medical University during 2011 to 2017. None of the enrolled patients had previously received preoperative chemotherapy or radiotherapy. Additionally, all patients enrolled in this study provided written informed consent, and the study protocol has been reviewed and approved by the Ethics Committee of the First Affiliated Hospital of Nanjing Medical University.

### 2.2 Cell Culture and Treatment

This study utilized human GC cell lines AGS, SNU‐484, HGC‐27, NCI‐N87, and MKN‐74, along with the human normal gastric epithelial cell line GES‐1. All cell lines were obtained commercially from the American Type Culture Collection (ATCC, USA). Cells were cultured in RPMI 1640 medium containing 10% fetal bovine serum (FBS; GIBCO, Brazil). The culture environment was a humidified incubator at 37°C with 5% CO_2_. For AGS and HGC‐27 cells, transfection was performed using Lipofectamine 2000 (Invitrogen, Carlsbad, CA, USA). Twenty‐four hours after transfection, the cells were harvested for subsequent experiments, and the sequences used in the experiments can be referred to in Additional file 1: Table S1. While circMTUS1‐si3 and ‐si4 were derived from the CircInteractome database, circMTUS1‐si1 and si2 were manually designed across the back‐spliced junction. To inhibit RNA and protein synthesis respectively, cells were treated with actinomycin D (Act D, CST, MA, USA) or cycloheximide (CHX, Selleck, TX, USA) for specified durations; additionally, MG132 (Selleck, TX, USA) was used to inhibit protein degradation through the proteasome pathway.

### 2.3 Lentiviral Infection and Stable Transfection Cell Line Construction

After counting, gastric cancer cells were uniformly seeded into 6‐well cell culture plates. Then the target virus was added for transduction. After transduction, the medium was replaced with fresh complete medium, and the cells were continued to be incubated in the 37°C, 5% CO_2_ incubator. 48 h later, the fluorescence intensity of cells was observed under a fluorescence microscope to verify the transduction efficiency. Subsequently, the medium was replaced with selection medium containing puromycin, and the cells were cultured continuously. During this period, the selection medium was replaced regularly to eliminate cells that failed to be transduced. Finally, stable gastric cancer cell lines with circMTUS1 overexpression or low expression were obtained.

### 2.4 RNA Extraction and Quantitative Real‐Time PCR (qRT‐PCR)

TRIzol reagent (Invitrogen, USA) was used for total RNA extraction from clinically collected tissues, and in vitro cultured cell lines. 500 ng of the qualified RNA was subjected to reverse transcription strictly following the standard protocol of the PrimeScript RT Reagent Kit (TaKaRa, China), and the resulting cDNA was stored at −20°C for later use. Using the synthesized cDNA as a template, the relative expression level of mRNAs was detected by qRT‐PCR. GAPDH was used to normalize the expression levels of circRNA and mRNA, thereby eliminating operational differences between samples. The specific primers used in this experiment were designed and synthesized by Nanjing Tsingke Biotechnology and detailed information of the primer sequences is provided in Additional file 1: Table S2. Finally, we calculated circMTUS1 expression fold changes across samples via the 2^−^^ΔCt^ method, with all experiments run in triplicate for reliability.

### 2.5 Nuclear‐Cytoplasmic Fractionation

NE‐PER reagents (Thermo Scientific, USA) were used following the manufacturer's instructions. Briefly, GC cells were lysed in protease inhibitor‐supplemented Lysis Buffer J on ice for 10 min, then centrifuged at 14 000×g for 3 min. The supernatant (cytoplasmic) and pellet (nuclear) fractions were processed for RNA isolation using Buffer SK. After purification, transcripts were analyzed by qRT‐PCR to assess their subcellular distribution.

### 2.6 Fluorescence In Situ Hybridization (FISH)

FISH was performed with specific probes targeting circMTUS1, following the protocol provided by GenePharma (Shanghai, China). Cells were fixed with 4% paraformaldehyde for 15 min at room temperature and subsequently rinsed with PBS. The washed cells were then combined with a solution for pre‐hybridization and probes. This combination allowed hybridization overnight at 37°C in a dark chamber with humidity control. After this process, cells were washed using saline‐sodium citrate buffer. Following washing, cells received incubation in blocking buffer for 1 h. This incubation was followed by overnight incubation with an antibody that contained HRP at 4°C. Finally, images were obtained and captured using a confocal microscope for visualization.

### 2.7 Protein Extraction and Western Blot Analysis

For protein extraction and Western blot analysis, proteins were extracted using radioimmunoprecipitation assay (RIPA) buffer. Supernatants that were derived from cell lysates received treatment using sodium dodecyl sulfate‐polyacrylamide gel electrophoresis on 10% acrylamide gels. Following this separation, transfer occurred onto a polyvinylidene difluoride (PVDF) membrane from Millipore. For western blot analysis, primary antibodies targeting PABPC1 (#ab21060, Abcam), LC3 (14600‐1‐AP, Proteintech), p62 (18420‐1‐AP, Proteintech), and YBX1 (20339‐1‐AP, Proteintech) were used, along with a horseradish peroxidase (HRP)‐conjugated secondary antibody (1:2000 dilution). Protein signals were visualized using a chemiluminescence‐based western blot detection system (Bio‐Rad).

### 2.8 Immunohistochemistry (IHC)

The procedure involved treating the samples with 4% formaldehyde solution at room temperature for 20 min, followed by a 30‐minute blocking treatment with 5% bovine serum albumin (BSA) in PBS buffer. Subsequently, primary incubation was performed. Overnight incubation at 4°C using PABPC1‐specific antibody (Abcam ab21060) and YBX1‐specific antibody (Proteintech 20339‐1‐AP). Then, the sections were incubated with an HRP‐conjugated secondary antibody for 1 h at room temperature. Protein expression was visualized using a 3,3'‐diaminobenzidine (DAB) kit, and nuclei were counterstained with hematoxylin. Images were captured using a light microscope.

### 2.9 Cell Counting Kit‐8 (CCK‐8) Assay

The experiment followed CCK8 (Dojindo Laboratories, Osaka, Japan) protocol. In the initial phase, AGS cells and HGC‐27 cells exhibiting typical growth patterns were seeded into a 96‐well plate at a density of 3×10^3^ cells per well. After the cells growing to a predetermined time point, 10 µL of CCK‐8 reagent was added to each well. The plates were then incubated at 37°C in a 5% CO_2_ environment for 1 h to complete the reaction required for the assay.

### 2.10 5‐Ethynyl‐2′‐Deoxyuridine (EdU) Incorporation Assay

To assess cell proliferative activity, the EdU assay was conducted with the Cell‐Light EdU Kit (RiboBio Co., Guangzhou, China). GC cells were seeded in 24‐well plates to 60%‐70% confluency, supplemented with EdU working solution (final concentration 50 µm). After 2 h incubation, cells were fixed with 4% paraformaldehyde, and then dark‐stained with Apollo staining solution, and counterstained with Hoechst 33342 (room temperature) for nuclear labeling. ImageJ software was used to count EdU‐positive (proliferation‐positive) and Hoechst‐positive (total) cells for proliferation rate calculation.

### 2.11 Colony Formation Assay

Cells were plated into 6‐well plates. The cultures were maintained for 14 days at 37°C. Subsequently, the cells were stained using a mixture of 0.1% crystal violet in 20% methanol, and the number of formed cell colonies was counted.

### 2.12 Migration and Invasion Assay

Transwell assays were utilized to assess cell migration and invasion. For the invasion assay, 100 µL of Matrigel (BD Bioscience, San Jose, USA) was precoated onto the chambers, followed by incubation for 30 min. Treated cells were resuspended and added to the upper chamber, while the lower chamber received 600 µL of complete medium. After incubating for 24–48 h, they were washed using PBS, followed by fixation with 4% paraformaldehyde and ultimately stained using 0.1% crystal violet. Randomly select 3 fields of view and count cells under an inverted fluorescence microscope.

### 2.13 Wound Healing Assay

After culturing the cells for 24 h, we used sterile 200 µL pipette tips to make scratches on the surface of monolayer cells. Following PBS washes, we switched the cells to serum‐free medium for another 24 h of incubation. Finally, we captured images of cell migration using an Olympus (Japan) inverted microscope and analyzed the results with ImageJ software.

### 2.14 Mouse Xenograft Model

Six‐week‐old female nude mice (from Laboratory Animal Center, Nanjing Medical University) were housed in SPF conditions. They received subcutaneous inguinal inoculation of 5×10^6^ GC cells (n = 5/group). Tumor volumes were measured every 4 days (volume = length×(width/2)^2^) and mice euthanized at 28 days for tumor harvest. For chemosensitivity testing, cisplatin (5 mg/kg in PBS) was ip injected thrice weekly, starting 1‐week post‐inoculation; xenografts were collected at 4 weeks. The study was approved by the Ethics Committee of First Affiliated Hospital, Nanjing Medical University.

### 2.15 RNA Immunoprecipitation (RIP)

The RIP experiment was conducted utilizing the Magna RIP RNA‐Binding Protein Immunoprecipitation Kit (Millipore), with procedures strictly adhering to the manufacturer's instructions. The o8G antibody, PABPC1 antibody, YBX1 antibody, and IgG antibody were used in the assay.

### 2.16 RNA Pull‐Down Assay

The biotin‐labeled circMTUS1 probe was specifically designed and synthetically produced for us by GenePharma (Shanghai, China). For probe immobilization, circMTUS1 probes were initially incubated with C‐1 magnetic beads (Life Technologies, Waltham, MA, USA) at 25°C for 2 h to facilitate bead surface coating. Subsequent to cell harvesting and lysis, the resultant lysates were co‐incubated with either circMTUS1 probes or oligonucleotide control probes at 4°C overnight. RNA‐bead complexes were isolated utilizing the RNeasy Mini Kit (Qiagen), and circMTUS1 expression were quantitatively determined by qRT‐PCR.

### 2.17 Flow Cytometry to Detect ROS Levels

We measured intracellular ROS with a Beyotime ROS assay kit (Cat. No. S0033S). First, we diluted the fluorescent probe DCFH‐DA 1:1000 in serum‐free medium for a 10 µm working solution. We added 1 mL to target cells, then incubated in the dark for 20 mins. Following the period of incubation, we conducted washing of cells three times using PBS to remove probe that did not show binding, applied treatment to separate cells, used a method of rotation to collect, and provided a final wash with PBS for purposes of making pure. We placed cells in suspension in 500 µL PBS and applied a Beckman Coulter (Miami, USA) device measuring flow to provide measures of signal from ROS, conducting assessment of ROS levels.

### 2.18 TUNEL Assay

We conducted the TUNEL assay following the specific approach that the producer indicates for the Servicebio kit (Servicebio, Wuhan, China). Cells were treated with Sclareol for 48 h, then received fixing in 4% paraformaldehyde at temperature of the room for 15 min, with treatment for making permeable using a buffer for this purpose for 10 min that followed. Following washing with PBS, the cells received incubation with the mixture for the reaction of terminal deoxynucleotidyl transferase (TdT) that provides measurement using signal at 37°C for 1 h. Following washing with PBS that occurred after this, the structures for covering received mounting to surfaces of glass using Medium for Mounting that contains DAPI from Vectashield Antifade (Beyotime, Shanghai, China). Images using signal received collection using a Nikon device for viewing with signal (Nikon Corporation, Tokyo, Japan) and received analysis with programs for processing images.

### 2.19 Cell Viability Analysis

Cells received placement in 96‐well plates for each well and received incubation for 24 h. Following this, they received treatment with different levels of agents, such as cisplatin, 5‐fluorouracil, doxorubicin hydrochloride, and mitomycin C, for 48 h. The measure of cells that remain living received assessment using an MTT Assay Kit (Sigma‐Aldrich, St. Louis, MO, USA) to conduct assessment of effects that cisplatin produces that relate to death of cells. The measure of absorption at 490 nm received assessment with a device for reading structures with multiple wells, and the measure of inhibition at half of maximum (IC50) received calculation.

### 2.20 Immunofluorescence

Cells received placement in confocal dishes and followed by treatment with substance for drugs. Immunofluorescence procedure included sequential fixation using 4% paraformaldehyde, permeabilization using Triton X‐100, and blocking using goat serum. After incubation with primary antibody along with the corresponding secondary antibody, cells were labeled with DAPI and finally captured via confocal laser scanning microscopy (Zeiss, Oberkochen, Germany).

### 2.21 o8G RIP

o8G RIP assays were performed using a o8G RIP kit (GenSeq, GS‐ET‐007). For RNA preparation, cells were lysed with TRIzol reagent to extract total cellular RNA; 50 µg of RNA was fragmentated to meet subsequent experimental requirements. Protein A/G beads conjugated with antibodies were prepared by dividing into two tubes: anti‐o8G antibody and IgG antibody were added respectively. For immunoprecipitation, input was stored at −20°C temporarily, the residual RNA was mixed with RIP buffer, aliquoted equally into the two tubes, and incubated on a vertical rotator at ambient temperature for 1 h. Subsequent to RNA elution, qPCR was performed to quantify circMTUS1 expression in each group, and the resultant qPCR amplicons were subjected to agarose gel electrophoresis analysis.

### 2.22 CLIP

CLIP testing is strictly conducted following the manufacturer's operating procedures using the CLIP kit (BersinBio, Bes3014). circMTUS1 truncation primers (50 bp in length; Sequences are detailed in Table S3) and reverse primers incorporating a plus‐tailed universal primer (PCR Reverse Primer) were custom‐designed. The cells were pre‐cultured for 16 h in a complete medium containing 10% FBS with 100 µm 4‐thiouracil (Sigma‐Aldrich, catalog no. #T4509, St. Louis, MO, USA) prior to subsequent experimental procedures. Antibody‐protein A/G bead preparation was conducted as follows: 2 µg of target antibody or isotype‐matched IgG control was added to protein A/G beads for the IP group or IgG control group, respectively, followed by incubation on a vertical rotator at 4°C. For UV crosslinking, 3×10^7^ cells were placed on ice and irradiated with 365 nm UV light for 10 min to stabilize protein‐RNA interactions. Post‐crosslinking, cells were lysed in 1× cell lysis buffer on ice for 10 min. Following immunoprecipitation, protein digestion and total RNA extraction were performed sequentially, and circMTUS1 enrichment was quantitatively assessed via qPCR.

### 2.23 Co‐IP

Co‐IP assays were performed strictly per the manufacturer's protocol using a Co‐IP Kit (BersinBio, Cat. #Bes3011) to investigate protein‐protein interactions. Antibody‐protein A/G beads were divided into IP and IgG control groups, followed by 30‐min incubation at room temperature. The protein extraction procedure was the same as previously described. For immunoprecipitation, 100 µL of the supernatant was reserved as input, while 600 µl aliquots were added to each group and incubated with shaking on a vertical rotator at 4°C for 1 h. Following elution of protein complexes with WB elution buffer, the supernatant was analyzed by polyacrylamide gel electrophoresis (PAGE) followed by western blotting.

### 2.24 Statistical Analysis

Data are presented as mean ± standard deviation (SD), with general statistical analyses performed using GraphPad Prism 9.5.1. For comparisons between two groups, an unpaired two‐tailed Student's t‐test was used. For comparisons among three or more groups, ordinary one‐way analysis of variance (ANOVA) or two‐way ANOVA was applied as appropriate. Two‐way repeated measures ANOVA was used when data involved both time and treatment factors. For datasets with unequal variances, Welch ANOVA was employed. Appropriate post hoc multiple comparison tests were applied where necessary. Survival curves were constructed via the Kaplan–Meier method, and intergroup differences were compared by the log‐rank test. A two‐tailed *p* < 0.05 was considered statistically significant.

## 3 Results

### 3.1 CircMTUS1 is Significantly Downregulated in Gastric Cancer Tissues and Cells

**FIGURE 1:**
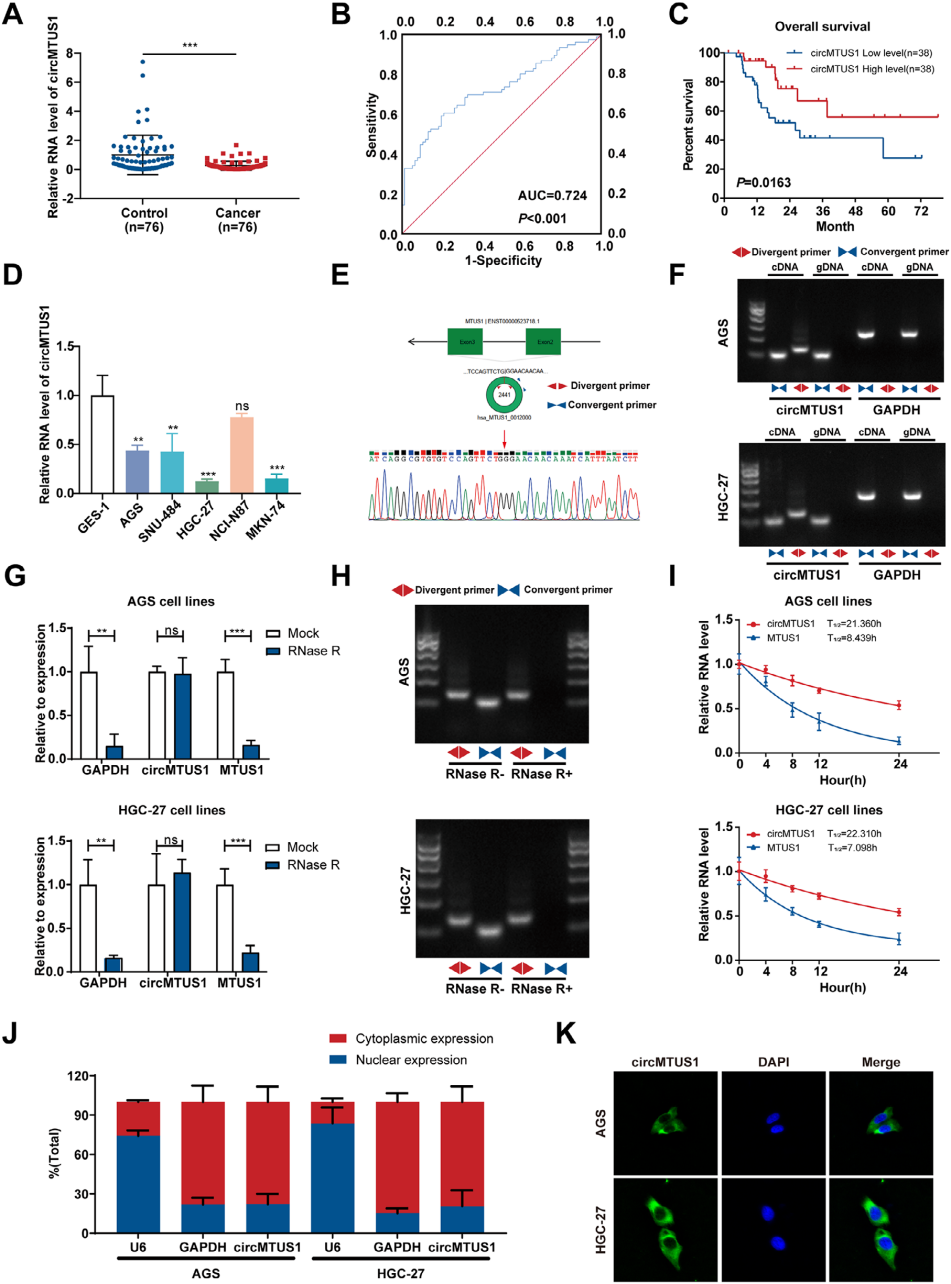
The expression and characteristics of circMTUS1 in GC tissues and cells. (A) qRT‐PCR of circMTUS1 expression in GC tissues. (B) The ROC curve to evaluate the diagnostic value of circMTUS1. (C) Kaplan‐Meier analysis of the correlation between circMTUS1 expression and overall survival in GC patients. (D) The expression of circMTUS1 in GC cells. E: Sanger sequencing of circMTUS1. The arrows represent splicing sites. (F,G) qRT‐PCR determining the abundance of circMTUS1 in AGS and HGC‐27 cell lines after RNase R treatment. (H) qRT‐PCR products of linear and circular products amplified with convergent and divergent primers with and without RNase R treatment. (I) qRT‐PCR analysis of circMTUS1 and MTUS1 after actinomycin D treatment. (J) qRT‐PCR of separated nuclear and cytoplasmic fractions. (K) FISH for circMTUS1 localization in AGS and HGC‐27 cell lines. Data are presented as mean ± SD from at least three independent experiments. ^*^ *p* < 0.05, ^**^ *p* < 0.01, ^***^ *p* < 0.001. One‐way ANOVA test (D), Two‐way ANOVA test (I), Student's t‐test (A,G), and log‐rank test (C) were used to determine statistical significance.

CircRNAs that play pivotal roles in gastric cancer (GC) progression were identified through the analysis of circRNA high‐throughput sequencing datasets (GSE100170, GSE184882, GSE152309, GSE248612) (Figure S1A–D). There were three circRNAs, which were significantly downregulated across four GEO datasets (Figure S1E). circMTUS1(hsa_circ_0083444) was selected for further study, which was most downregulated in GC compared with paired noncancerous tissues (Figure 1A and Figure S1F,G). Clinicopathological parameters were analyzed according to circMTUS1 expression in GC tissues (Table 1). We found that reduced circMTUS1 expression correlated with advanced GC (stage III + IV), lymph node metastasis, and increased tumor invasion depth (Figure S2A–D). No correlation was noticed between circMTUS1 RNA level and other clinical variables, including gender, age, histological subtype, and tumor dimension (Figure S2E–H). Moreover, the receiver operating characteristic (ROC) curve revealed the diagnostic efficacy of circMTUS1 in GC through an area under the curve (AUC) of 0.724 (*p* < 0.001) (Figure 1B). Kaplan‐Meier survival analysis indicated that GC patients with lower circMTUS1 expression exhibited inferior overall survival outcomes (*p* = 0.0163) (Figure 1C). To investigate whether circMTUS1 exerts a role in gastric cancer carcinogenesis, we detected the expression of circMTUS1 in cells and found its expression was also notably downregulated in GC cell lines (Figure 1D). Meanwhile, we found circMTUS1 consisted 2441‐nucleotides originated from back‐splicing of the MTUS1 gene, which was verified by Sanger sequencing, and the sequencing results aligned with circBase database annotations (Figure 1E). Furthermore, circMTUS1 was amplifiable with divergent primers using cDNA, but not with genomic DNA (Figure 1F), and was resistant to RNase R (Figure 1G,H). Then we used actinomycin D (ActD) to treat AGS and HGC‐27 cells to detect RNA stability and the results indicated that circMTUS1 exhibited a longer half‐life and higher stability compared with its homologous linear mRNAs (Figure 1I). Analysis of nuclear and cytoplasmic RNA via qRT‐PCR (Figure 1J) and fluorescence in situ hybridization (FISH) targeting circMTUS1 (Figure 1K) demonstrated its preferential cytoplasmic localization. Collectively, these data suggested that circMTUS1, characterized by stability and predominant cytoplasmic localization, may be implicated in GC pathogenesis.

**TABLE 1:** Association Between circMTUS1 expression and clinicopathological characteristics in gastric cancer.

Characteristics	N	High expression	Low expression	P
**Gender**				
**Female**	22	9	13	0.8450
**Male**	54	29	25	
**Age(y)**				
**≤60**	33	18	15	0.2998
**>60**	43	20	23	
**Tumor size(cm)**				
**>5**	51	29	22	0.7772
**≤5**	25	9	16	
**Histological type**				
**Well differentiated**	41	24	17	0.3607
**Poorly differentiated**	35	14	21	
**Clinical stage**				
**I‐II**	34	30	4	<0.001***
**III‐IV**	42	8	34	
**Tumor depth**				
**T1‐T2**	21	16	5	0.0285*
**T3‐T4**	55	22	33	
**Lymph node metastasis**				
**No**	17	14	3	0.0064**
**Yes**	59	24	35	

### 3.2 CircMTUS1 Significantly Inhibits Gastric Cancer Progression In Vitro

**FIGURE 2:**
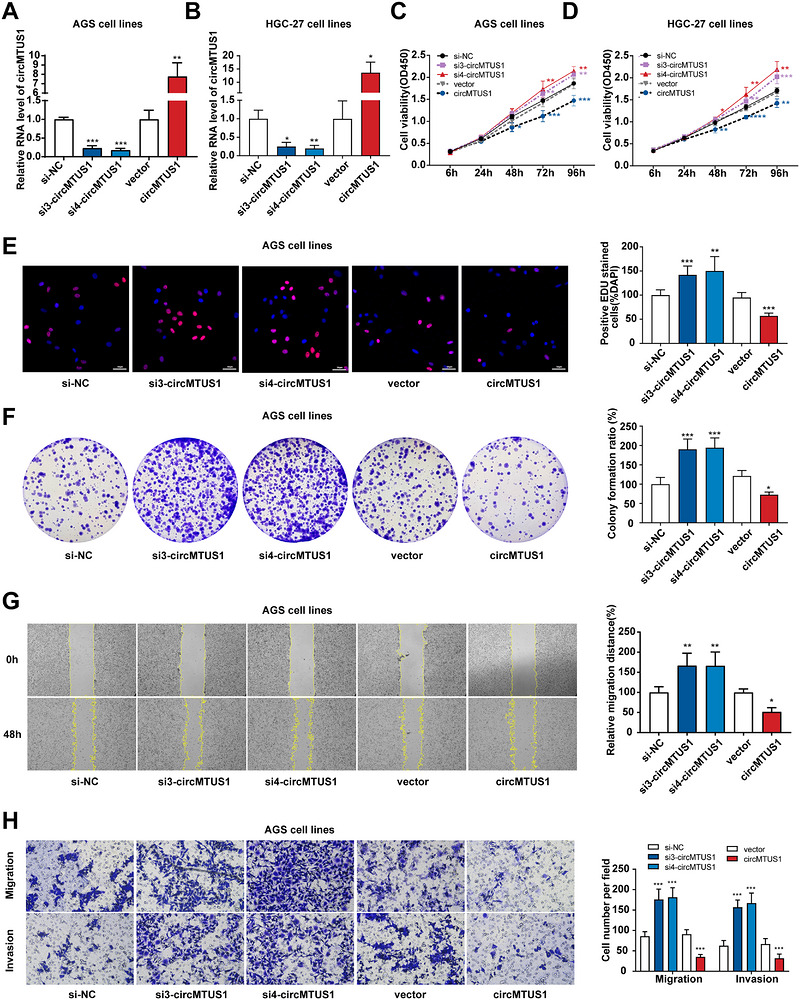
circMTUS1 suppresses the malignant transformation of GC cells. (A,B) qRT‐PCR analysis of circMTUS1 expression after treatment with siRNAs and an overexpression plasmid. (C,D) Assessment of the proliferation of AGS and HGC‐27 cells transfected with circMTUS1‐specific siRNA or an overexpression plasmid by a CCK‐8 assay. (E,F) Assessment of AGS cell proliferation by EdU(E) and colony formation assays (F). (G) Wound healing assay to detect the effect of circMTUS1 on cell migration. (H) Decreased or increased circMTUS1 regulated the migration and invasion of AGS cells. Data are presented as mean ± SD from at least three independent experiments, ^*^ *p* < 0.05, ^**^ *p* < 0.01, ^***^ *p* < 0.001. One‐way ANOVA test (A, B, E, F, G, H), Two‐way ANOVA test (C,D), and Student's t‐test (A,G) were used to determine statistical significance.

To investigate the biological role of circMTUS1 in GC, we generated circMTUS1‐specific siRNAs and overexpression plasmids, and validated their efficiency (Figure 2A,B and Figure S3A). Subsequent in vitro functional assays were performed to characterize its biological functions: CCK‐8 assay showed that knockdown of circMTUS1 notably promoted the viability of GC cells, while its overexpression clearly reduced such viability (Figure 2C,D). EdU assays showed that circMTUS1 silencing enhanced the proliferation of GC cells, whereas its overexpression significantly suppressed proliferation (Figure 2E and Figure S3B,C). Colony formation assays revealed that overexpression of circMTUS1 significantly impaired the colony‐forming potential of GC cells, whereas its downregulation elicited the converse effect (Figure 2F and Figure S3D). As GC progresses, advanced‐stage patients often develop metastases to multiple organs, which poses a severe threat to their life and health. The Transwell and wound healing assays revealed that circMTUS1 restrained GC cell migration and invasion (Figure 2G,H and Figure S3E,F). To rule out the effect of linear MTUS1, we first detected that circMTUS1 overexpression had no impact on MTUS1 (Figure S4A,B). Then, after overexpressing MTUS1 (Figure S4C,D), we found that cell proliferation and migration were not significantly enhanced (Figure S4E–L). Collectively, these findings further indicated that circMTUS1 could markedly impede GC progression in vitro.

### 3.3 CircMTUS1 Significantly Inhibits Gastric Cancer Progression In Vivo

**FIGURE 3:**
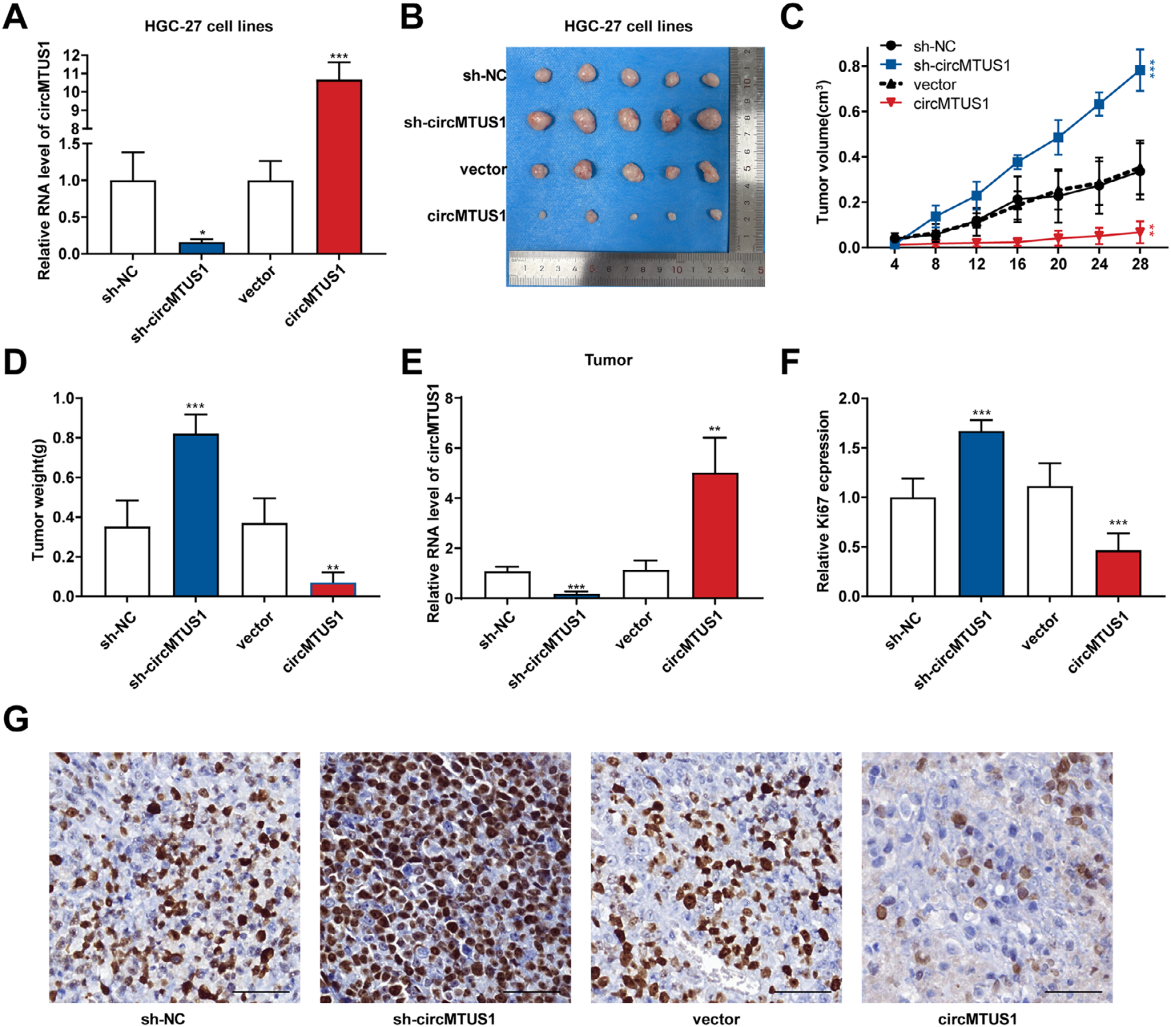
circMTUS1 significantly inhibits gastric cancer progression in vivo. (A) Stable overexpression and low‐expression efficiency of circMTUS1 in lentivirus‐infected HGC‐27 cells. (B) Tumor growth curves for each group of nude mice during rearing. (C) Excision of the tumors for photographic documentation. (D) Weights of the tumors from the nude mice. (E) Expression of circMTUS1 in tumors of each group. (F,G) Immunohistochemical analysis and quantitative analysis of Ki67 expression. Data are presented as mean ± SD from at least three independent experiments, ^*^ *p* < 0.05, ^**^ *p* < 0.01, ^***^ *p* < 0.001. One‐way ANOVA test (A, D, E, F) and Two‐way ANOVA test (C) were used to determine statistical significance.

To determine whether circMTUS1 inhibits GC progression in vivo, we established a subcutaneous xenograft model in nude mice. Stable circMTUS1‐overexpressing or knockdown HGC‐27 cell lines were constructed through lentiviral transduction (Figure 3A). In comparison with the control group, tumors in circMTUS1 overexpression group were smaller, whereas those in the circMTUS1 knockdown group were larger (Figure 3B). Twenty‐eight days after tumor formation, the nude mice were euthanized, and the tumors were resected for subsequent observation and weighing. In comparison to the control group, tumors in circMTUS1‐overexpressing group exhibited reduced size (Figure 3C) and decreased weight (Figure 3D). The expression level of circMTUS1 in tumor tissues exhibited a negative correlation with tumor size (Figure 3C–E). The Immunohistochemical (IHC) analysis was performed to evaluate the expression of Ki67, a well‐established proliferation‐related biomarker. Relative to the control group, tumors in the circMTUS1 overexpression group exhibited decreased Ki67 expression, whereas higher Ki67 expression was observed in the circMTUS1 shRNA group (Figure 3F,G). These data confirmed that circMTUS1 could notably suppress GC progression in vivo.

### 3.4 CircMTUS1 o8G Modifications

**FIGURE 4:**
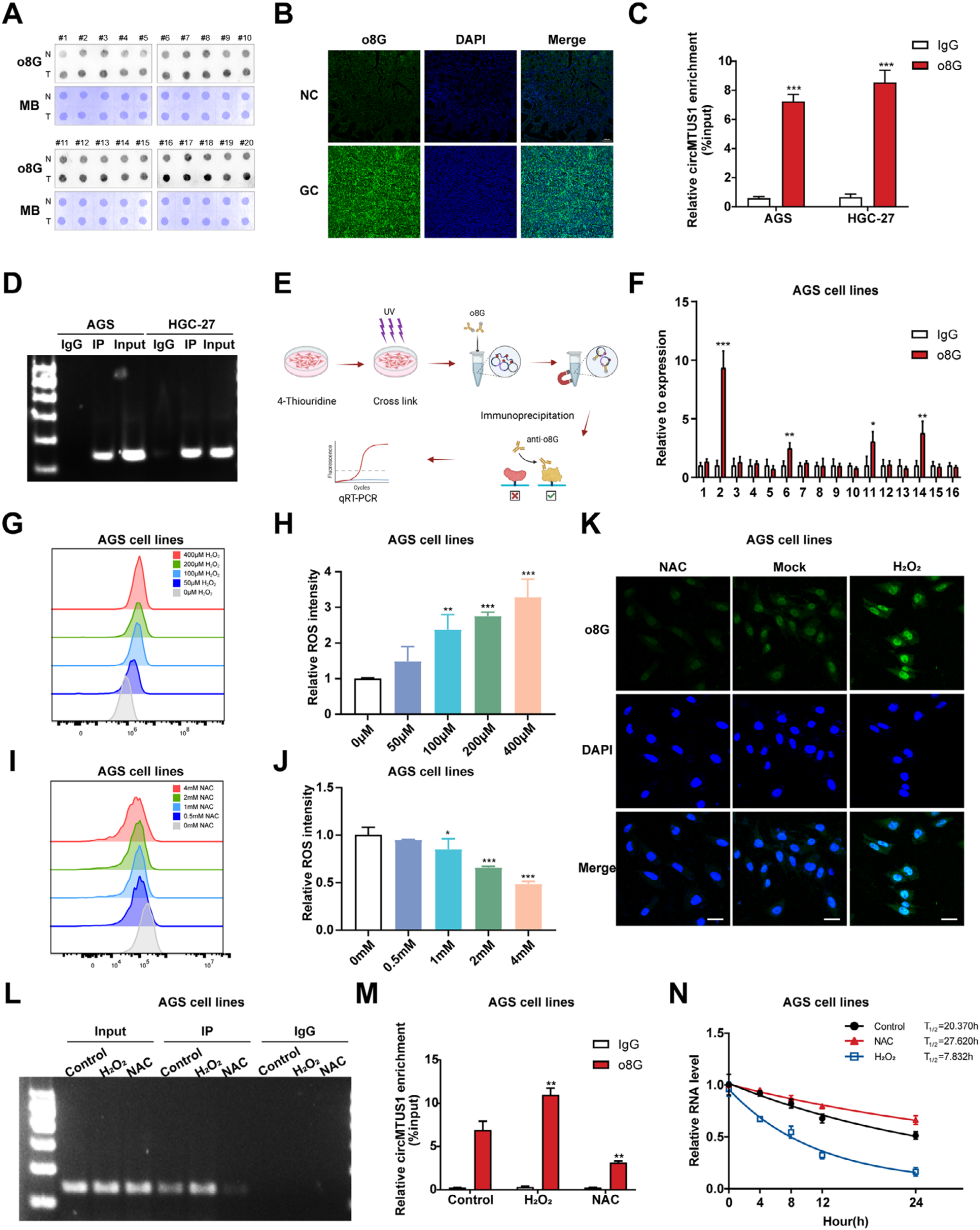
circMTUS1 o8G modification. (A) o8G dot blot assay of global o8G abundance in GC patients. MB staining is used as a loading control. (B) o8G expression in GC tissues tested by IF. (C,D) Results of circMTUS1 o8G RIP‐qPCR and agarose gel electrophoresis. (E) Schematic diagram of the CLIP experiment. (F) CLIP‒qPCR analysis of the o8G modification region in circMTUS1 in AGS cell lines. G‐J and H: Intracellular ROS detection in AGS cells treated with different concentrations H_2_O_2_ (G) and NAC (I). Statistical analysis of ROS levels after treatment with different concentrations of H_2_O_2_ (H) and NAC (J). (K) IF staining of o8G modification levels after treatment with H_2_O_2_ and NAC in AGS cell lines. (L) The RIP products were subjected to agarose gel electrophoresis after treatment with H_2_O_2_ and NAC. (M) RIP assay and qPCR analysis of circMTUS1 o8G modification after treatment with H_2_O_2_ and NAC. (N) An RNA stabilization assay was carried out after treatment of AGS cells with H_2_O_2_ and NAC. Data are presented as mean ± SD from at least three independent experiments, ^*^ *p* < 0.05, ^**^ *p* < 0.01, ^***^ *p* < 0.001. One‐way ANOVA test (H, J), Two‐way ANOVA test (M, N), and Student's t‐test (C, F) were used to determine statistical significance.

With the deepening of research on RNA modifications, the critical roles of o8G modification in regulating RNA molecular fate, alongside its involvement in tumorigenesis and progression, have been elucidated [16, 20]. Targeted dot blot screening across gastric cancer and paired normal tissues identified o8G as the candidate with the most significant differential expression, a pattern further validated in cell lines (Figure 4A and Figure S5A–D). IF and IHC staining validated a prominent elevation in o8G expression within GC tissues (Figure 4B and Figure S5E). o8G RIP assay was performed to confirm the presence of o8G modification on circMTUS1 by using an o8G specific primary antibody, and the data demonstrated substantial enrichment of circMTUS1 by anti‐o8G antibody (Figure 4C,D). To map the o8G modification region(s) of circMTUS1, specific primers were designed to amplify truncated circMTUS1 fragments in CLIP assays. We demonstrated that o8G modification was predominantly localized to segments 2, 6, 11, and 14 of circMTUS1 (Figure 4E,F and Figure S5F). To further investigate whether the o8G modification of circMTUS1 was triggered by the attack of reactive oxygen species (ROS) [15, 21], we employed concentration gradients of hydrogen peroxide (H_2_O_2_) and N‐acetylcysteine (NAC) to assess intracellular ROS levels to treat GC cell lines. Ultimately, a 400 µm H_2_O_2_ concentration (Figure 4G,H and Figure S5G,H) and a 4 mm NAC concentration (Figure 4I,J and Figure S5I,J) were chosen for subsequent experimental procedures. We subsequently performed IF, which showed that H_2_O_2_ elevated intracellular o8G modification levels while NAC reduced them (Figure 4K and Figure S5K). Accordingly, H_2_O_2_‐treated cells were subjected to o8G RIP assays. Results revealed elevated o8G modification of circMTUS1 in H_2_O_2_‐treated groups and reduced modification in NAC‐treated groups, which confirmed that ROS‐induced o8G modification of circMTUS1 (Figure 4L,M and Figure S5L,M). Moreover, this ROS‐induced modification shortened circMTUS1 half‐life and diminished its stability (Figure 4N and Figure S5N).​ These observations indicated that o8G modification existed on circMTUS1, and there was a positive correlation between the level of o8G modification and the intracellular ROS level.

### 3.5 CircMTUS1 Interacts With YBX1 and PABPC1 in GC Cells

**FIGURE 5:**
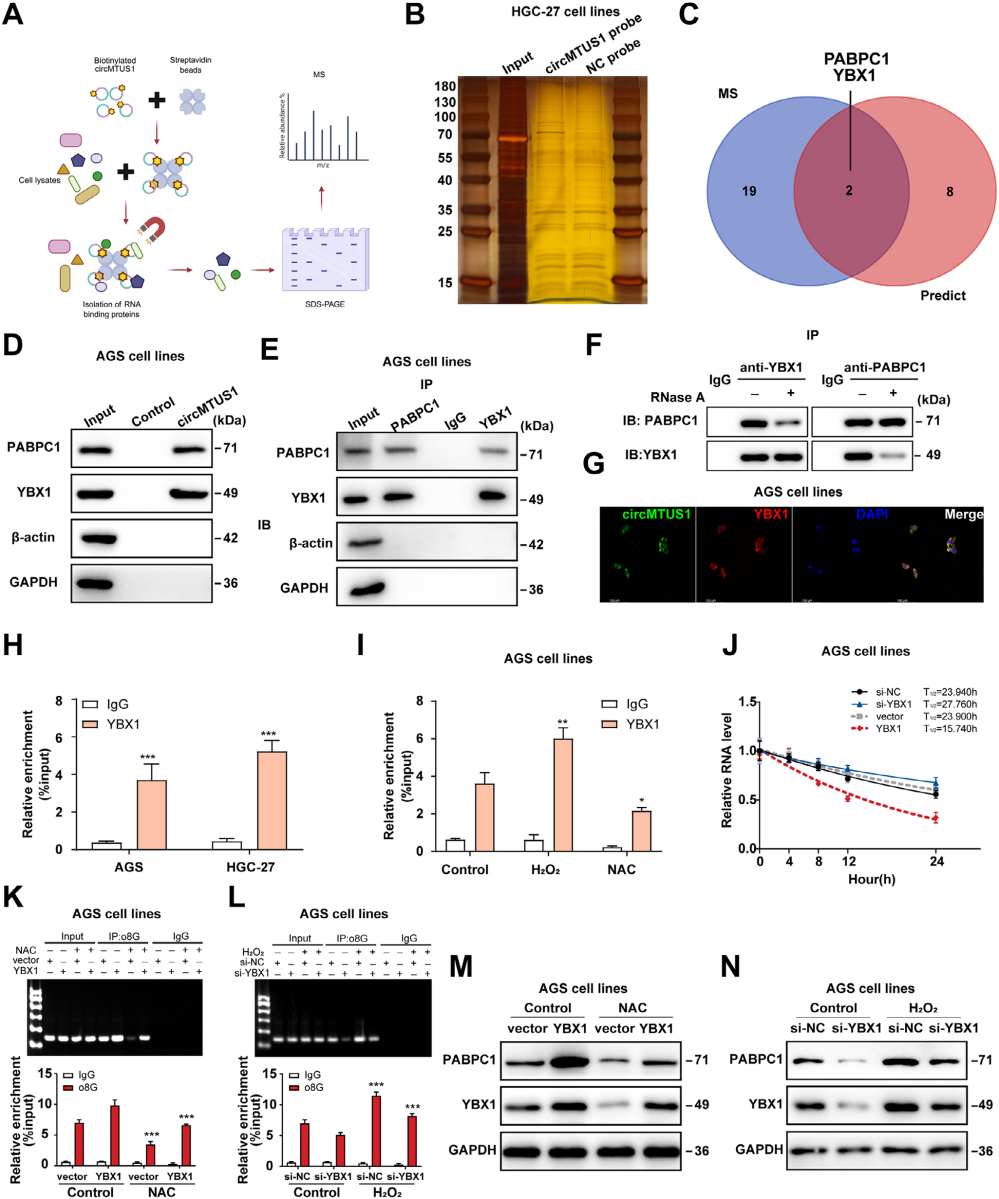
YBX1 modulated o8G of circMTUS1. (A) Schematic diagram of the circMTUS1 pulldown experiment. (B) circMTUS1 probes and control probes were biotinylated and incubated with AGS cell lysates for RNA pull‐down assays and silver staining for the proteins precipitated in the RNA pull‐down assays. (C) circMTUS1 binding proteins detected by RNA pull‐down and mass spectrometry combined with bioinformatics prediction. (D) Immunoblotting analysis of YBX1 and PABPC1 in RNA pull‐down samples by circMTUS1 probes and control probes in AGS cell lines. (E) Co‐IP followed by immunoblotting analysis of YBX1 and PABPC1 in AGS cells. (F) The interaction between YBX1 and PABPC1 weakened after circMTUS1 was digested with RNase A. (G) The co‐localization of circMTUS1 with YBX1 in AGS cell lines is detected using FISH combined with immunofluorescence assays. (H) A RIP‒qPCR assay was performed to detect the interaction between circMTUS1 and YBX1. (I) YBX1 RIP assay and qPCR analysis of circMTUS1 after treatment with H_2_O_2_ and NAC. (J) An RNA stabilization assay was carried out after treatment of AGS cells with YBX1 siRNA or an overexpression plasmid. (K,L) o8G‐RIP qPCR analysis of YBX1 overexpression or depression after treatment with H_2_O_2_ or NAC. (M,N) Overexpress and knockdown YBX1 in cells treated with H_2_O_2_ or NAC, and detect the protein expression of YBX1 and PABPC1. Data are presented as mean ± SD from at least three independent experiments, ^*^ *p* < 0.05, ^**^ *p* < 0.01, ^***^ *p* < 0.001. One‐way ANOVA test (I, K, L), Two‐way ANOVA test (J), and Student's t‐test (H) were used to determine statistical significance.

To clarify the molecular mechanism underlying the carcinostatic effect of circMTUS1 in GC, we conducted a biotin‐labeled RNA pull‐down assay with circular junction‐targeted probes in GC cells, followed by mass spectrometry (MS) (Figure 5A). qRT‐PCR analysis in junction‐specific probe pull‐down precipitates showed higher enrichment of circMTUS1 than the control probe, but not MTUS1 mRNA (Figure S6A,B). Following SDS‐PAGE combined with silver staining of RNA pull‐down precipitates, specific protein bands were identified in the circMTUS1 pull‐down samples (Figure 5B and Figure S6C,D). From these protein bands, 21 proteins were identified in HGC‐27 cells via MS analysis (Figure 5C), among which 2 overlapped with the predicted proteins (Figure 5C and Figure S6E). Then, RNA pull‐down assays confirmed that PABPC1 and YBX1 were highly enriched by the circMTUS1 probe in both AGS and HGC‐27 cells (Figure 5D and Figure S6F). PABPC1‐YBX1 interaction was verified via Co‐IP (Figure 5E and Figure S6G). To explore the role of circMTUS1 in this interaction, we treated the complex with RNase A (which digests circMTUS1) and observed a weakening of the YBX1‐PABPC1 interaction (Figure 5F). FISH combined with immunofluorescence demonstrated cytoplasmic colocalization of circMTUS1 and YBX1, which implies an interaction of them (Figure 5G and Figure S6H). In addition, YBX1‐specific antibodies were shown to significantly enrich circMTUS1 relative to control IgG antibodies via RIP assays (Figure 5H). Notably, YBX1 was previously identified as an RNA modification reader such as m5C or o8G [22, 23]. However, we failed to detect any significant enrichment of circMTUS1 when we performed m5C RIP assays (Figure S6I). To investigate whether YBX1 regulates circMTUS1 stability through recognizing o8G modification, we conducted YBX RIP‐qPCR and found that H_2_O_2_ increased the binding of YBX1 to circMTUS1, whereas NAC exerted the opposite effect (Figure 5I and Figure S6J). In actinomycin D decay assays, YBX1 overexpression accelerated the degradation of circMTUS1, while YBX1 knockdown prolonged its half‐life (Figure 5J and Figure S6K). Knockdown of YBX1 significantly reduced the o8G‐bound circMTUS1 level, whereas YBX1 overexpression increased it (Figure S6L). Under oxidative stress induced by H_2_O_2_, the level of o8G‐bound circMTUS1 was markedly increased, and this effect was reversed by YBX1 siRNA. Conversely, treatment with NAC reduced o8G‐bound circMTUS1, and this reduction was counteracted by YBX1 overexpression, indicating that YBX1 is required for the recognition of o8G‐modified circMTUS1 (Figure 5K,L and Figure S6M,N). WB experiments using the aforementioned treated cells showed that siYBX1 decreased PABPC1 and could reversed by H_2_O_2_, while YBX1 enhanced PABPC1 may rescued by NAC (Figure 5M,N and Figure S6O,P). These data demonstrated that PABPC1, another protein that bound to circMTUS1, might also be regulated by the circMTUS1 which was mediated by YBX1. Collectively, these findings illustrated that YBX1 and PABPC1 were associated with circMTUS1, and that YBX1 served as an o8G modification reader to mediate the attenuation of circMTUS1 stability.

### 3.6 CircMTUS1 Binds to PABPC1, Preventing its Degradation Through Ubiquitination

**FIGURE 6:**
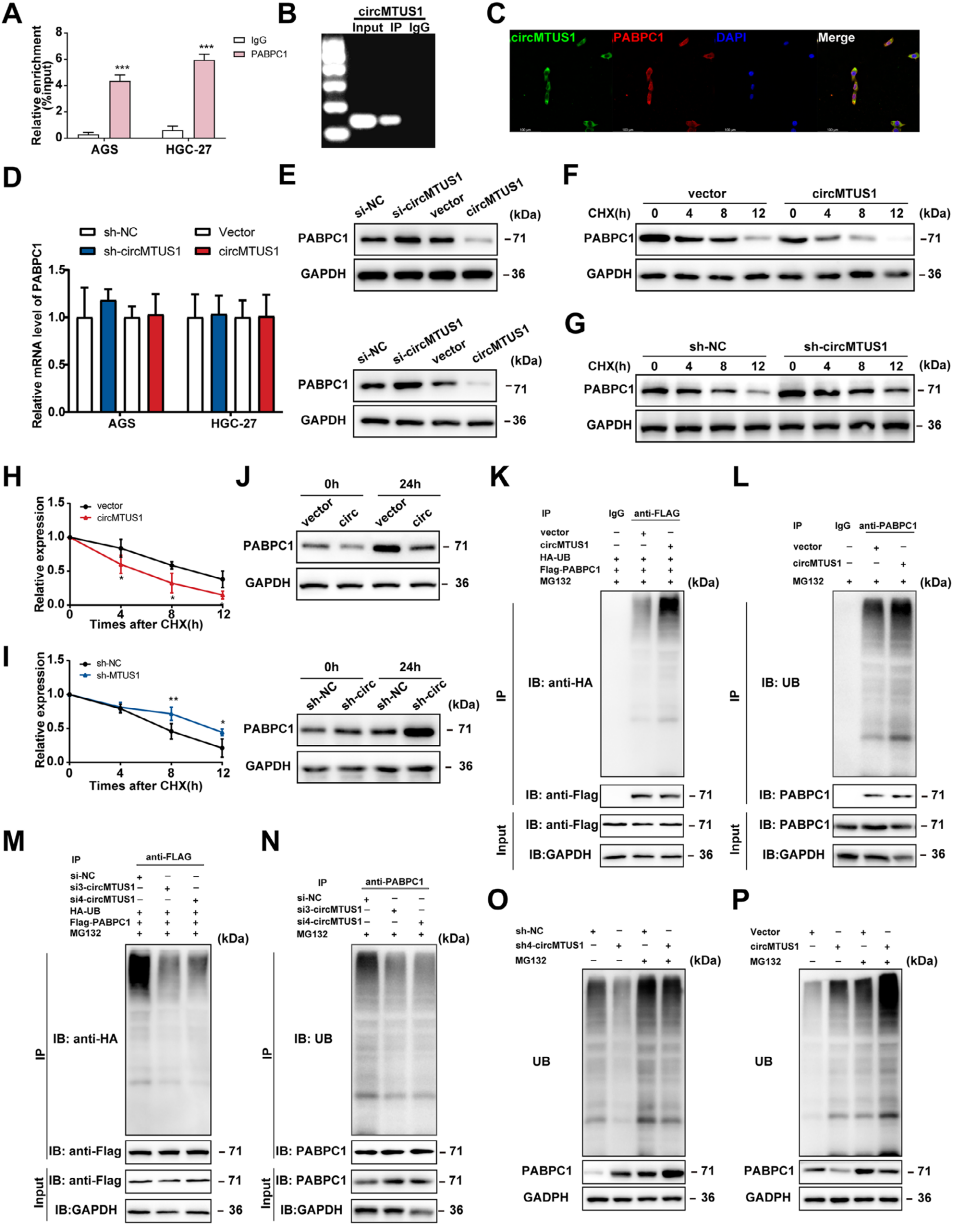
circMTUS1 binds to PABPC1, preventing its degradation through ubiquitination. (A,B) A RIP‒qPCR assay was performed to detect the interaction between circMTUS1 and PABPC1. (C) The co‐localization of circMTUS1 with PABPC1 in AGS cell lines is detected using FISH combined with IF assays. (D,E) The impact of circMTUS1 on PABPC1 RNA(D) and protein expression levels(E). (F,G) The expression levels of PABPC1 protein in different transfected cells. The cells were treated with cycloheximide (CHX, 100 µg/mL) for the indicated time points before harvesting. (I) The effects of MG‐132 on PABPC1 protein stability in different transfected cells. (J) IB analysis of lysates from cells transfected with HA‐Ub, Flag‐PABPC1 with vector or circMTUS1 plasmid. (K) IB analysis of lysates from cells transfected with vector or circMTUS1 plasmid. (L) IB analysis of lysates from cells transfected with HA‐Ub, Flag‐PABPC1 with NC or si3‐circMTUS1 or si4‐circMTUS1. (M) IB analysis of lysates from cells transfected with NC or si3‐circMTUS1 or si4‐circMTUS1. All the cells(J–M) were treated with MG‐132 for 8 h before harvesting. (N,O) The effect of circMTUS1 on PABPC1 ubiquitination was evaluated in cells treated with or without MG132. Data are presented as mean ± SD from at least three independent experiments, ^*^ *p* < 0.05, ^**^ *p* < 0.01, ^***^ *p* < 0.001. One‐way ANOVA test (H, I) was used to determine statistical significance.

Subsequently, RIP assays were performed with a PABPC1‐specific antibody, and found the binding of circMTUS1 to PABPC1 (Figure 6A,B and Figure S7A). FISH combined with IF confirmed the colocalization of circMTUS1 and PABPC1 in the cytoplasm of GC cells, which further provided evidence for their interaction (Figure 6C and Figure S7B). To investigate their regulatory interplay, we assessed the impact of circMTUS1 on PABPC1 mRNA and protein expression levels. qRT‐PCR revealed that neither circMTUS1 knockdown nor overexpression altered PABPC1 mRNA levels (Figure 6D). However, circMTUS1 overexpression suppressed PABPC1 protein expression, whereas circMTUS1 downregulation enhanced PABPC1 protein expression, and altering circMTUS1 expression did not affect YBX1 levels (Figure 6E and Figure S7C). It has been well documented that PABPC1 undergone ubiquitin‐proteasome system (UPS)‐mediated degradation, which constituted the principal pathway for the turnover of intracellular proteins in vivo [24]. Furthermore, inhibition of protein synthesis using CHX revealed that PABPC1 protein levels were stabilized upon circMTUS1 inhibition, but destabilized following circMTUS1 overexpression (Figure 6F–I). Importantly, the proteasome inhibitor MG132 restored PABPC1 levels in cells with high circMTUS1 expression, indicating that circMTUS1 promoted PABPC1 degradation via the proteasome pathway (Figure 6J). Additionally, ubiquitination assays revealed that circMTUS1 silencing diminished PABPC1 ubiquitination levels, while circMTUS1 overexpression exerted opposing effects (Figure 6K–P). Meanwhile, data showed that YBX1 knockdown significantly increased the ubiquitination level of PABPC1, while YBX1 overexpression reduced PABPC1 ubiquitination. Under oxidative stress induced by H_2_O_2_, PABPC1 ubiquitination was markedly decreased. Conversely, NAC treatment increased PABPC1 ubiquitination (Figure S7D,E). Moreover, validated by CCK‐8, EdU, and Transwell assays, PABPC1 siRNA reversed the enhancement of circMTUS1 overexpression on GC cell proliferation and migration (Figure S8). These results provided evidence that circMTUS1 facilitated the ubiquitination‐coupled proteasomal degradation pathway of PABPC1.

### 3.7 CircMTUS1 Regulates the Sensitivity of GC Cells to Cisplatin

**FIGURE 7:**
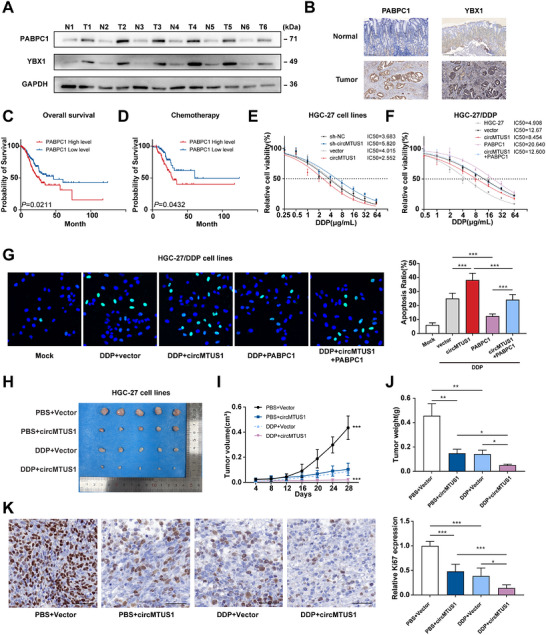
circMTUS1 regulates the sensitivity of GC cells to cisplatin. (A) The protein level of YBX1 and PABPC1 in GC tissues detected by WB. (B) The protein level of YBX1 and PABPC1 in GC tissues detected by IHC. (C,D) Kaplan‐Meier analysis of the correlation between PABPC1 expression and OS (C) or OS with chemotherapy (D). (E) Relative viability of stable circMTUS1 overexpression or knockdown HGC‐27 cells exposed to cisplatin at the indicated concentrations (0.25, 0.5, 1, 2, 4, 8, 16, 32, and 64 µg/mL)) for 48 h. (F) Cell viability of HGC‐27 and HGC‐27/DDP (cisplatin‐resistant) cells after circMTUS1/PABPC1 overexpression exposed to cisplatin at the indicated concentrations for 48 h. (G) TUNEL assays of HGC‐27/DDP cell lines cotransfected with PABPC1 and circMTUS1 overexpression plasmids. (H) Xenograft tumors of sacrificed mice treated with or without cisplatin at the experimental endpoint. (I,J) Tumor volumes (I) and weight (J) of nude mouse tumors. (K) The Ki67 expression in mice tumor tissues. Data are presented as mean ± SD from at least three independent experiments, ^*^ *p* < 0.05, ^**^ *p* < 0.01, ^***^ *p* < 0.001. One‐way ANOVA test (G, J, K), Two‐way ANOVA test (E, F, I), and log‐rank test (C, D) were used to determine statistical significance.

Regarding clinical significance, we continued to conduct WB and IHC to determine YBX1 and PABPC1 expression levels, and the results revealed a substantial upregulation of them in GC tissues compared with control tissues (Figure 7A,B). Given that YBX1 and PABPC1, which are positioned in the upstream and downstream of the circMTUS1 signaling pathway, could be correlated with GC prognostic characteristics [25, 26]. TCGA data revealed that higher expression levels of YBX1 and PABPC1 correlated with overall survival (OS) either alone or in combination with chemotherapy (Figure 7C,D and Figure S9A,B). Collectively, these results implied that circMTUS1 might contribute to the development of chemoresistance in GC cells.

Accordingly, cisplatin (DDP), 5‐fluorouracil (5‐FU), doxorubicin (DOX), and mitomycin C (MMC) were screened to determine which chemotherapeutic agent exhibited the greatest circMTUS1‐mediated modulation of sensitivity. The results demonstrated that upregulated circMTUS1 mostly inhibited the viability of cisplatin‐treated cells, whereas downregulated circMTUS1 contributed to the induction of cisplatin resistance (Figure 7E and Figure S9C–I). Moreover, circMTUS1 siRNA led to an increase in the half maximal inhibitory concentration (IC50), while circMTUS1 overexpression resulted in a decrease in IC50 (Figure 7E and Figure S9I). Functional assays revealed that while circMTUS1 overexpression alone impaired cisplatin resistance and promoted cell apoptosis, PABPC1 overexpression conversely enhanced drug resistance and suppressed apoptosis. Notably, concurrent overexpression of PABPC1 effectively reversed the cisplatin hypersensitivity induced by circMTUS1 (Figure 7F,G and Figure S9J,O). In vivo, we found that elevated circMTUS1 expression significantly inhibited the growth of xenograft tumors and enhanced the sensitivity of tumor cells to cisplatin treatment (Figure 7H–J). WB analysis revealed that upregulated circMTUS1 led to a significant reduction in PABPC1 protein levels (Figure S9P). Further IHC analysis of xenograft tumor samples indicated that Ki67 expression was significantly decreased following circMTUS1 overexpression (Figure 7K). Moreover, results showed that DDP treatment remarkably increased the o8G modification level of circMTUS1 and reduced circMTUS1 expression. Importantly, NAC largely abolished these alterations induced by DDP (Figure S9K–N). Taken together, these results confirmed that circMTUS1 governed the sensitivity of GC cells to cisplatin through PABPC1.

### 3.8 PABPC1 Can Form Granules and Exhibit Liquid‐Like Features

**FIGURE 8:**
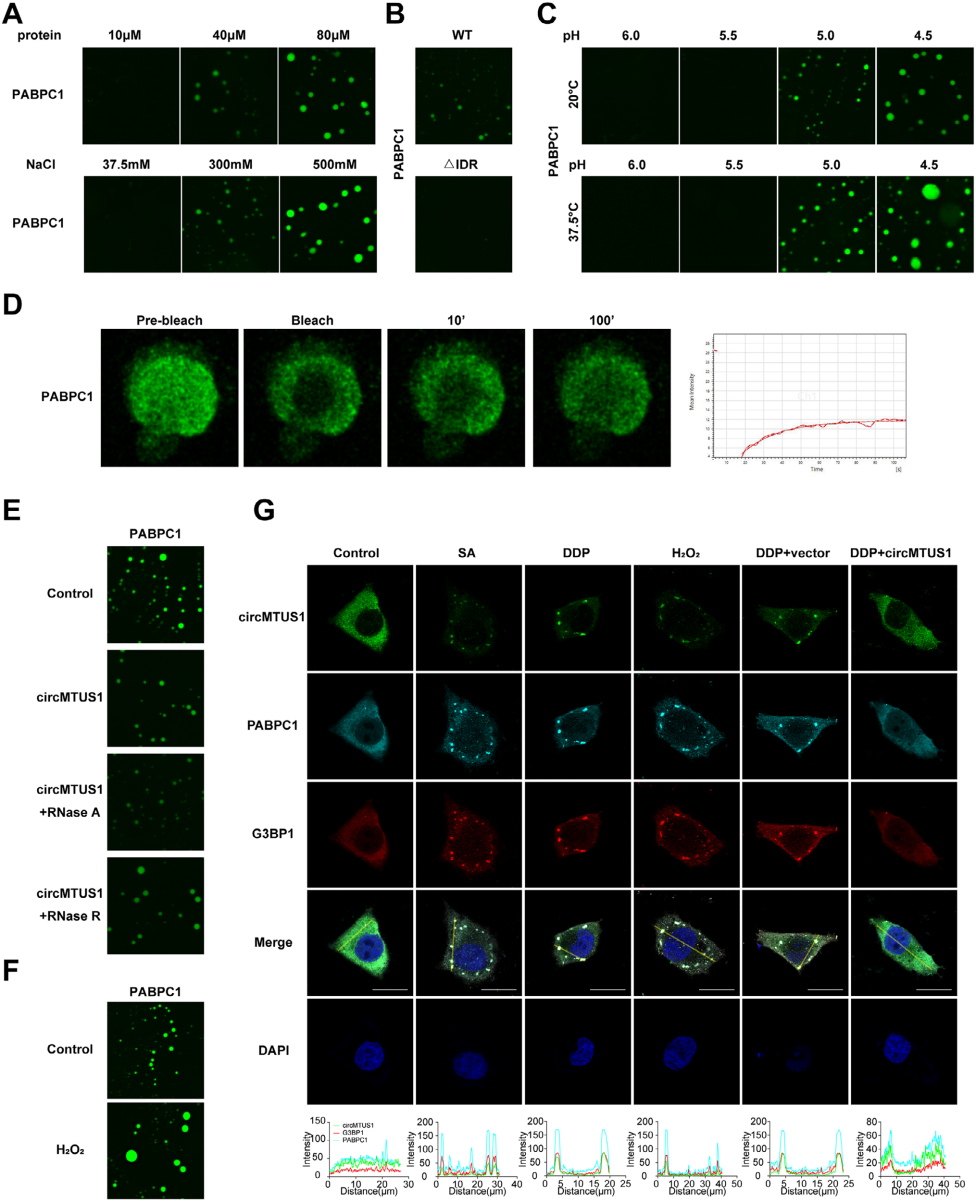
PABPC1 can form granules and exhibit liquid‐like features. (A) GFP fluorescence to analyze GFP‐PABPC1 droplet formation at different protein concentration(upper) and salt ion concentrations(lower) at room temperature. (B) The effect of absence of GFP‐PABPC1 on droplet formation after IDR region deletion in vitro.(C) Droplet formation results of GFP‐PABPC1 at different pH value and temperature. (D) Fluorescence recovery after photobleaching (FRAP) of GFP‐PABPC1 droplets. (E) In vitro LLPS of purified PABPC1‐GFP with circMTUS1 with or without different types of RNase. (F) In vitro LLPS of purified PABPC1‐GFP with H_2_O_2_. (G) Immunofluorescence indicating circMTUS1 (green), G3BP1(Red) and PABPC1 (Cyan) in AGS cells. The cells were incubated with SA/H_2_O_2_/DDP and transfected with circMTUS1 plasmid.

Emerging evidence has demonstrated that intrinsically disordered regions (IDRs) serve as essential structural prerequisites for protein phase separation, a process that contributes to tumorigenesis by modulating transcriptional regulation, gene expression, RNA splicing, and other cellular processes [27]. Bioinformatic analysis enabled the detection of a notable IDR within the amino acid sequence of PABPC1 (Figure S10A). Previous research has implicated PABPC1 as an essential mediator of SG formation, and SG may be generated in the cellular cytoplasm under stress conditions via liquid‐liquid phase separation (LLPS) [28]. GO functional enrichment analysis of proteins captured with circMTUS1 via RNA pulldown showed that SG may be involved in circMTUS1‐driven gastric carcinogenesis (Figure S10B). Purified PABPC1 promoted droplet formation, where small droplets merged into larger assemblies in a salt‐ and concentration‐dependent manner (Figure 8A). Nevertheless, no droplet formation was detectable following deletion of the IDR (Figure 8B), indicating that PABPC1 mediated LLPS through its intrinsic IDR under in vitro conditions. Droplet formation showed a dependence on both temperature and pH, with a significant enlargement of droplet size as the temperature rose from 20°C to 37°C (Figure 8C). Additionally, after photobleaching, purified GFP‐PABPC1 proteins underwent recovery, further validating the dynamic characteristics of PABPC1‐mediated phase separation (Figure 8D). In addition, to identify the influence of circMTUS1 on droplet formation of PABPC1, we transcribed and cyclized circMTUS1 to incubate with PABPC1 protein. We found that circMTUS1 could promote droplets of PABPC1, and this facilitatory effect was attenuated by RNase A while showing no response to RNase R, implying that circMTUS1 was involved in mediating the formation of PABPC1 droplets (Figure 8E). Also, we found that treatment with H_2_O_2_ significantly promoted the punctate aggregation and LLPS formation of the PABPC1 protein (Figure 8F). Chemotherapy‐stimulated cells might acquire drug resistance through the formation of SG [29], of which PABPC1 was a critical component. Consequently, we further investigated the role of circMTUS1 in regulating SG formation in cisplatin‐treated cells. Confocal microscopy revealed that under basal conditions, endogenous PABPC1 was diffusely distributed in the cytoplasm. However, acute exposure to DDP uniformly triggered PABPC1 to condense into cytoplasmic puncta that co‐localized with G3BP1. Furthermore, circMTUS1 overexpression effectively dissolved these G3BP1/PABPC1‐positive stress granules (Figure 8G and Figure S10C). The above results showed that circMTUS1 might inhibit SG formation by regulating the LLPS of PABPC1.

### 3.9 The Small Molecule ISRIB Targeting SG Greatly Enhanced DDP Sensitivity in Synergy With CircMTUS1

**FIGURE 9:**
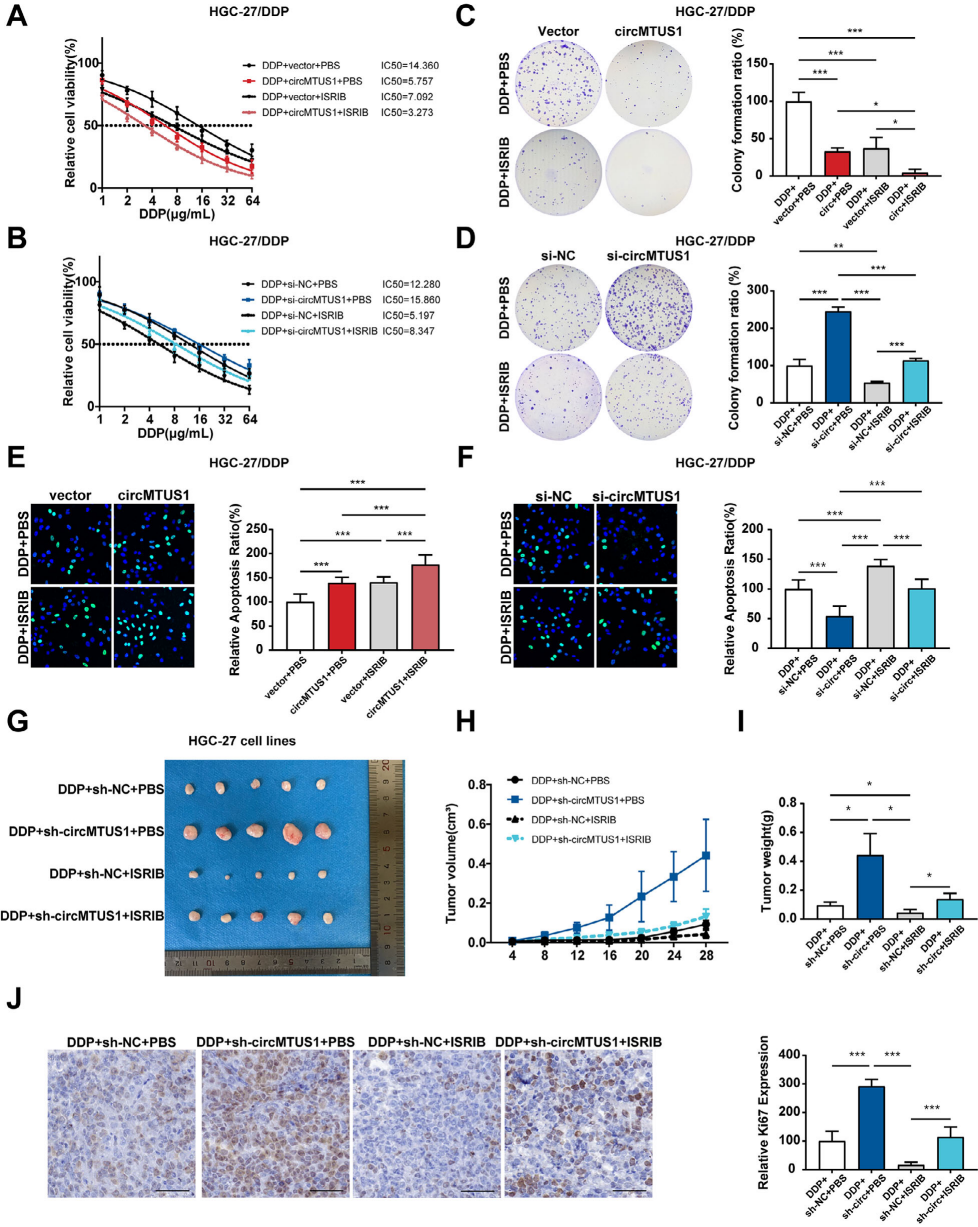
ISRIB exerted synergistic roles with circMTUS1 in improving chemosensitivity of GC cells. (A,B) Chemosensitivity to DDP of HGC‐27/DDP cells treated with circMTUS1 overexpression plasmid or si‐circMTUS1, ISRIB alone or in combination(A). (C,D) Colony formation assays showed the synergistic roles of circMTUS1 and ISRIB in increasing cell chemosensitivity. (E,F) TUNEL assays showed the synergistic roles of sh‐circMTUS1 and ISRIB in increasing cell chemosensitivity. For chemosensitivity testing, cisplatin (5 mg/kg in PBS) was ip injected thrice weekly, starting 1‐week post‐inoculation; xenografts were collected at 4 weeks. (G) Images of xenograft tumors after the indicated treatment. (H,I) Growth curves and relative weights of the xenograft tumors in the four groups. The group treated with PBS was used as a control. (J) IHC staining and statistics for Ki67 in the xenograft tumors of different groups. Data are presented as mean ± SD from at least three independent experiments, ^*^ *p* < 0.05, ^**^ *p* < 0.01, ^***^ *p* < 0.001. One‐way ANOVA test (C, D, E, F, I, J) and Two‐way ANOVA test (A, B, H) were used to determine statistical significance.

It has been confirmed that the small molecule ISRIB could effectively reverse eIF2α phosphorylation‐mediated effects and induce SG disassembly [30]. Our study showed that the combined use of the circMTUS1 overexpression plasmid and ISRIB further enhanced the DDP sensitivity of GC cells. Conversely, ISRIB significantly mitigated the chemoresistance induced by circMTUS1 knockdown (Figure 9A,B). Colony formation and TUNEL assays provided additional corroboration for these results (Figure 9C–F). For in vivo assessment of ISRIB's function in chemoresistance, nude mice were subcutaneously implanted with cells that had been stably transfected with sh‐circMTUS1. Treatment with sh‐circMTUS1 alone could accelerate tumor growth, while ISRIB administration improved the response to DDP by mitigating DDP resistance (Figure 9G–I). Ki67 staining of xenograft tumor tissues indicated that circMTUS1 knockdown continued to promote cell proliferation under DDP treatment, while this proliferation was reduced following ISRIB treatment (Figure 9J). These findings implied that ISRIB represented a potential therapeutic agent that could be utilized in combination with chemotherapy for GC treatment.

### 3.10 Autophagy is Involved in CircMTUS1‐Mediated DDP Resistance

**FIGURE 10:**
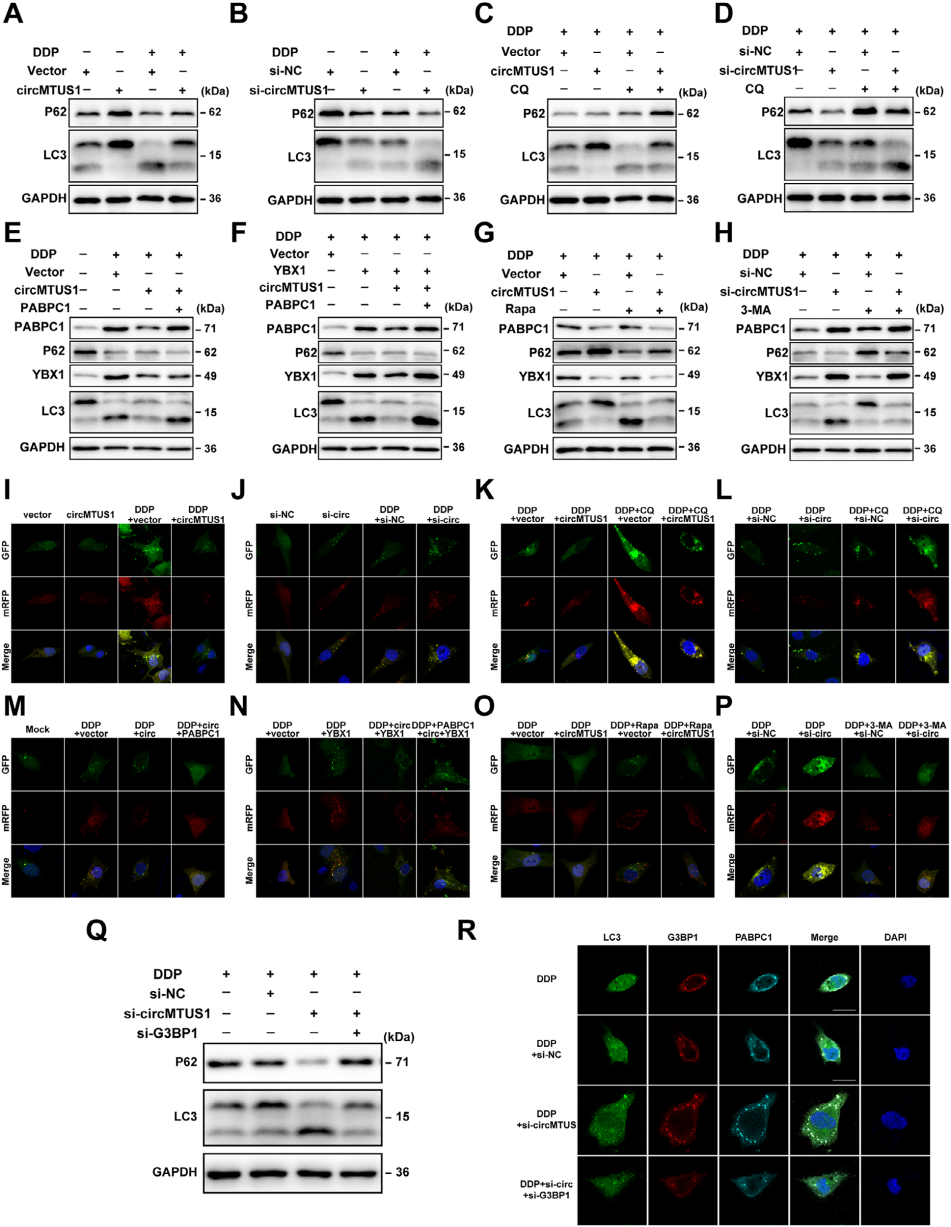
Autophagy is involved in circMTUS1‐mediated DDP resistance. (A,B) Expression levels of P62 and LC3B‐I/II in indicated cells with or without DDP treatment by western blotting. (C,D) Western blotting performed on the cisplatin‐resistant HGC‐27 cells (HGC‐27/DDP) 24 h after treatment with DDP combined with or without CQ (20 µm) against LC3B‐/II and P62. (E) HGC‐27 and HGC‐27/DDP cells were treated with the following conditions, mock, DDP+ vector, DDP+circMTUS1 overexpression plasmid, and DDP+circMTUS1+PABPC1, to detect PABPC1 and autophagy‐related proteins (P62 and LC3B‐I/II). (F) HGC‐27/DDP cells were treated with YBX1, circMTUS1, PABPC1 plasmid alone or in combination to detect YBX1, PABPC1, and autophagy‐related proteins (P62 and LC3B‐I/II). (G) Expression levels of P62 and LC3B‐I/II in HGC‐27/DDP cells treated with RAPA (20 µm) plus vector and circMTUS1 plasmid detected by western blotting. (H) Expression levels of LC3B‐I/II and P62 in the cells treated with 3‐MA (2 mm) plus si‐NC and si‐circMTUS1 detected by western blotting. (I–P) GFP and mRFP double‐positive dots (Green+ Red+; yellow in the merged images) represent autophagosomes, where LC3 is associated with the phagophore before fusion with lysosomes. mRFP single‐positive dots (Green‐ Red+) indicate autolysosomes, where GFP fluorescence is quenched in the acidic lysosomal environment, but mRFP fluorescence remains stable. (Q) Expression levels of LC3B‐I/II and P62 in the HGC‐27/DDP cells treated with si‐G3BP1 plus si‐circMTUS1 detected by western blotting. R: Immunofluorescence indicating LC3 (green), G3BP1(Red) and PABPC1 (Cyan) in HGC‐27/DDP cells. The cells were incubated with DDP and transfected with si‐circMTUS1 and si‐G3BP1. Data are presented as mean ± SD from at least three independent experiments, ^*^ *p* < 0.05, ^**^ *p* < 0.01, ^***^ *p* < 0.001.

Previous investigations have shown that autophagy regulated the chemoresistance of GC cells to DDP [31], and autophagy was involved in SG clearance [32, 33]. To explore the potential correlation between circMTUS1 and autophagy, we analyzed two classic autophagy markers: LC3B and P62. The results demonstrated that under DDP‐induced stress, circMTUS1 knockdown decreased the P62 level, together with the corresponding LC3B‐I to LC3B‐II transition that derived autophagy progression, whereas circMTUS1 overexpression had the opposite effect (Figure 10A,B). Next, cells were treated with either circMTUS1 siRNA or an overexpression plasmid together with chloroquine (CQ), which hinders autophagic flux and leads to the buildup of LC3B‐II. The results demonstrated that a notable accumulation of LC3B‐II occurred post si‐circMTUS1 treatment. Further evidence showed that reduced circMTUS1 activated autophagy, which could be inhibited by CQ, was evidenced by the accumulation of P62 after CQ treatment (Figure 10C). At the same time, the opposite impact was induced by circMTUS1 overexpression (Figure 10D). Thus, circMTUS1 expression exerted a suppressive effect on autophagy in GC cells, which was implied by these results. Given that circMTUS1 enhanced the sensitivity of gastric cancer cells to cisplatin, it is worthwhile to investigate whether circMTUS1 regulates DDP sensitivity by inhibiting autophagy via PABPC1. WB analysis verified that circMTUS1 induced a reduction in PABPC1 expression and the LC3 II/I ratio, and this reduction was rescued by the PABPC1 overexpression plasmid. Additionally, the suppressive effect of circMTUS1 on P62 was reversed by the overexpression of PABPC1. Notably, the upstream protein YBX1 appeared to remain unaffected by alterations in circMTUS1 and PABPC1 expression (Figure 10E). Consequently, upon adding YBX1 overexpression to the aforementioned treatments, we found that YBX1‐promoted autophagy was reversed by circMTUS1, and this reversal was further restored by PABPC1 (Figure 10F). For deeper investigation, cells were subjected to treatment with rapamycin (RAPA, an autophagy agonist) and 3‐methyladenine (3‐MA, an autophagy inhibitor). WB analysis showed that RAPA promoted autophagy in vector cells. However, in circMTUS1 overexpressed cells, the promotional effect of RAPA was attenuated, which further suggested that autophagy activation could be restrained by circMTUS1 expression (Figure 10G). 3‐MA, inhibited autophagy in circMTUS1‐knockdown cells in a similar manner (Figure 10H). Moreover, the results of autophagosome‐related puncta (GFP+ RFP+ and GFP– RFP+) analysis were consistent with the findings from WB experiments (Figure 10I–P). This cross‐validation between WB and IF strengthened the conclusion that circMTUS1 plays a critical role in autophagy modulation. To determine whether SG are necessary for PABPC1 degradation and whether this process occurs within autophagosomes, we performed functional blockade and colocalization analyses. WB showed that while DDP treatment combined with circMTUS1 knockdown activated autophagy, simultaneous depletion of G3BP1 significantly abrogated this autophagy induction (Figure 10Q). Furthermore, multi‐color immunofluorescence imaging revealed that punctate PABPC1 aggregates strictly co‐localized with G3BP1 and the autophagy marker LC3 (Figure 10R). This visual triple‐colocalization confirms that PABPC1 is sequestered into autophagosomes. Collectively, these results suggested that the progression of autophagy in GC cells was effectively inhibited by circMTUS1 through PABPC1.

## 4 Discussion

In recent years, circRNAs have become a key focus of academic research [34]. Advances in high‐throughput sequencing technologies have not only enabled the identification of more circRNAs but also deepened our understanding of mechanisms of these RNA molecules. In addition, growing evidence has shown that abnormal circRNA expression plays a vital role in cancer development and progression [35]. Gastric cancer (GC) is one of the most common cancers globally, with high rates of morbidity and mortality, placing an especially heavy health burden on East Asian nations [36]. While chemotherapies and immunotherapies for GC have advanced significantly in recent clinical practice, postoperative recurrence and distant metastasis remain major unresolved challenges [37]. Therefore, exploring the drivers of GC and devising strategies to overcome chemoresistance is essential for improving clinical outcomes. In the present study, we observed downregulation of circMTUS1 in GC tissues and GC cell lines, implying that circMTUS1 might function as a regulatory factor in GC progression. Additionally, we demonstrated that up‐regulated circMTUS1 suppressed the proliferation, migration, invasion, and chemoresistance of GC cells. However, it has been reported that circMTUS1 may act as an oncogene in conjunctival melanoma to modulate tumor‐associated pathways [38]. The discrepancies in the expression pattern and functional role of circMTUS1 are presumably associated with the tissue‐specific characteristics of circRNAs.

As a type of oxidative modification involved in cellular stress responses, the o8G modification was initially discovered in genomic DNA [20]. While studies on RNA o8G modification remain limited, this scarcity is attributed to the inherent rapid turnover of most RNA molecules, which limits the detection and analysis of modified RNA species. Nevertheless, circRNAs often exhibit even greater metabolic persistence due to their non‐linear, closed‐loop structure. This unique structural advantage establishes a compelling foundation for exploring the prevalence and functional significance of o8G modifications in circRNAs [14, 15]. During tumorigenesis, environmental stressors and pathophysiological stimuli disrupt ROS homeostasis, resulting in dysregulated o8G modification levels. Abnormal buildup of o8G modifications further affects disease progression [16, 39]. Here, we found that o8G modifications induced by ROS accumulation disrupted the stability of circMTUS1 and promoted GC progression. This aligns with previous research that redox imbalance and abnormal o8G modifications exist in different types of tumors [40, 41].

Furthermore, to validate whether o8G modification regulates circMTUS1, we utilized o8G “readers” for its precise modulation [42, 43]. Drawing on MS data and subsequent experimental evidence, we focused on YBX1, which encodes a highly conserved cold‐shock domain protein featuring an extensive array of nucleic acid‐binding regions. Previous studies showed that YBX1 participated in diverse cellular events, such as DNA repair [44], transcriptional regulation [45], and mRNA processing and stabilization [46]. Furthermore, YBX1 was previously reported to interact with m5C‐modified mRNAs to enhance mRNA stability and affect the expression levels of the downstream proteins [47]. According to Liu's research, circTMEM45A interacts with the m5C readers ALYREF and YBX1. These interactions facilitate the nuclear export and stability of NLRP3 mRNA [48]. Li et al. demonstrated that by binding and stabilizing YBX1, the lncRNA FGD5‐AS1 suppresses ROS‐induced senescence to accelerate GC progression [49]. Zhu's research revealed the regulatory significance of PPM1B‐driven YBX1 dephosphorylation and USP10‐driven YBX1 deubiquitination in the modulation of PANoptosis and oxaliplatin sensitivity in GC [26]. Mechanistically, YBX1 may act as a protective reader to indirectly sustain the steady‐state abundance of o8G modifications originally induced by ROS stress, which was consistent with the research of Zheng et al. [50]. Although the specific eraser targeting circMTUS1 remains unidentified in this study and represents a limitation to be addressed, the novel role of YBX1 in recognizing o8G to promote transcript decay remains robust.

The functional regulation of circRNAs mainly relies on their subcellular distribution. Nuclear‐resident circRNAs usually influence gene expression by affecting transcriptional activation [51] or splicing events [52]. On the other hand, cytoplasmic circRNAs primarily function as miRNA sponging [53] or interacting with proteins [54]. CircRNAs that bind to proteins have drawn wide attention, usually exerting effects as protein sponges or structural scaffolds for protein complexes. As shown in Qin et al.’s work, hsa_circ_0038737 reinforced DNPH1 mRNA stability through its interaction with IGF2BP3 [55]. As reported in Yao et al.’s study, circRNA‐mTOR regulated the nuclear translocation of RNA‐binding proteins, including PC4 and PSIP1 to promote cancer progression and enhanced its resistance to lenvatinib in hepatocellular cancer [56]. In the present research, circMTUS1 showed a predominant cytoplasmic localization, while PABPC1 was confirmed as an interactor of circMTUS1 through the combined use of RNA pulldown, MS, and RIP assays. PABPC1 is a member of the poly(A)‐binding protein family, which plays an important role in regulating gene expression and is involved in tumorigenesis. Zhang et al. previously reported that PABPC1 was increased in hepatocellular cancer and promoted cell proliferation [57]. Another study by An's group showed that PABPC1 was upregulated in GC. Elevation of PABPC1 showed an inverse correlation with patients’ overall survival and disease‐free survival [58]. In another study by Zhang et al., circSTX6 was identified to bind to PABPC1, leading to higher expression levels of SUZ12, which mediated DDP resistance in bladder cancer [59]. Building on the observed overexpression of PABPC1 in GC, our work identified a novel interaction between circMTUS1 and PABPC1. We further revealed that circMTUS1 exerts a tumor‐suppressive role by enhancing the ubiquitination and subsequent reduction of PABPC1 protein.

LLPS is a process that occurs when proteins or RNA aggregate at high concentrations to form membrane‐less condensates. LLPS is crucial for maintaining the orderly progression of core biological events, including cellular signaling, cell cycle regulation, protein activity modulation, and chromatin structural remodeling [60]. Recent studies have expanded the regulatory repertoire of circRNAs to include the modulation of LLPS, underscoring their diverse roles in cellular organization. For example, circRNF13 can bind to IGF2BP1, and the interaction between circRNF13 and IGF2BP1 suppressed ubiquitin‐mediated degradation of IGF2BP1 while promoting the assembly of its phase‐separated aggregates. This discovery offered new insights into the pathogenesis and progression of oral cancer [19]. Research found that under various environmental stresses such as chemotherapy exposure, heat shock, oxidative stress, osmotic stress, and nutrient deprivation, untranslated mRNA and proteins can form SG through LLPS [61]. For instance, circVAMP3 has been shown to inhibit the proliferation and migration of hepatocellular carcinoma cells. Mechanistically, circVAMP3 facilitates the formation of SG by driving CAPRIN1‐mediated LLPS [61]. SG, as dynamic biological cellular structures, play a critical role in supporting cell survival under adverse microenvironmental conditions and further contribute to the acquisition of chemoresistance in cancer cells. Our mechanistic investigation further demonstrated that the small‐molecule modulator ISRIB can block circMTUS1‐mediated SG assembly. In this study, circMTUS1 ablation neutralized the inhibitory effect of ISRIB on GC cisplatin resistance. This occurs because circMTUS1 deficiency stabilizes the downstream scaffold PABPC1, thereby bypassing the upstream SG blockade imposed by ISRIB to maintain SG‐mediated chemoresistance. Notably, the therapeutic efficacy of ISRIB has been previously documented in independent studies focusing on prostate cancer [62] and acute myeloid leukemia, highlighting its potential as a chemosensitizing agent [63]. Furthermore, this study represents an investigation to clarify ISRIB's role in DDP resistance in GC models, thereby enriching current chemotherapeutic sensitization strategies for GC.

Moreover, evidence indicated that dysregulation of autophagy associated with tumor adaptation served as a key mechanism underpinning chemotherapy resistance in cancer cells [64]. Notably, recent studies have proven that autophagy dysfunction constituted a potential mechanism contributing to the development of chemotherapy resistance [65]. In line with this, our results confirmed that autophagy was activated in DDP treated GC cells. Similarly, circRNAs have been identified as regulators of autophagy. Fu et al. demonstrated that circSEC24B activated autophagy and induced chemoresistance in colorectal cancer by promoting the deubiquitination of SRPX2, a process mediated by the deubiquitinating enzyme OTUB1 [66]. Furthermore, Liu et al.’s research broadened the knowledge of SG homeostasis regulation by showing that TRIM21 and autophagy receptors participated in modulating SG formation and clearance, which provided insights for developing therapeutic approaches for neurodegenerative diseases [32]. Ouyang et al. showed that G3BP1 deficiency exacerbates metabolic stress‐induced liver injury by impairing autophagic lipid degradation [67]. Consistent with the regulatory role of circRNAs in autophagy, our study found that overexpression of circMTUS1 decreased autophagy levels and attenuated chemotherapy resistance in GC cells.

## 5 Conclusion

**FIGURE 11:**
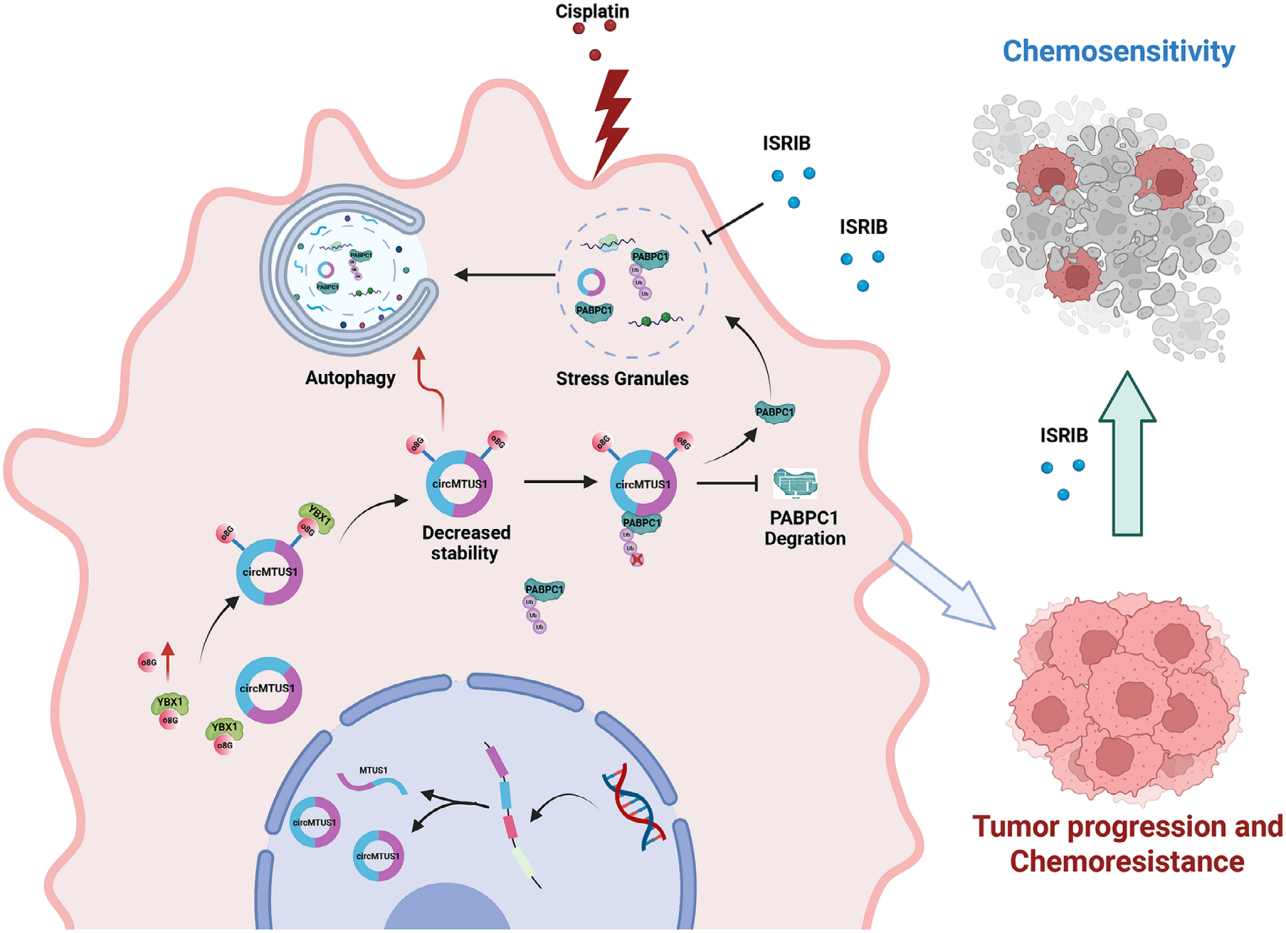
Schematic illustration of the mechanism.

Notably, we characterized circMTUS1, a circRNA derived from the back‐splicing of MTUS1 exons 2 and 3, that was implicated in GC. Beyond its capacity to promote the tumorigenic behaviors of GC cells, the association between low circMTUS1 levels and poor survival outcome highlighted its utility as a prognostic predictor. Mechanistically, YBX1 served as a reader protein recognizing the o8G modification on circMTUS1, a process that significantly compromised the stability of the circular RNA. Our study revealed that circMTUS1 bound to PABPC1 and regulated its ubiquitination and LLPS propensity, thereby modulating SG dynamics and autophagy in response to DDP (Figure 11). In summary, our study verified that circMTUS1 was a potential diagnostic and prognostic biomarker for GC. Therefore, strategies targeting circMTUS1 and SG formation might serve as viable approaches to improve chemosensitivity of GC.

## Author Contributions

**Lei Peng**: Conceptualization; Data curation; Formal analysis; Funding acquisition; Investigation; Resources; Supervision; Validation; Visualization; Writing – original draft; Writing – review & editing; **Lurong Li**: Data curation; Investigation; Software; Validation; Visualization; Writing – original draft; Writing – review & editing; **Zhenghui Zhu**: Data curation; Investigation; Writing – original draft; Writing – review & editing; **Chenyang Li**: Formal analysis; Funding acquisition; Investigation; Software; Visualization; Writing – original draft; Huaimin Sang: Formal analysis; Investigation; Software; Writing – review & editing; **Guoxin Zhang**: Methodology; Writing – review & editing; **Xuan Li**: Conceptualization; Project administration; Resources; Supervision; Validation; Writing – original draft; Writing – review & editing; **Yini Dang**: Methodology; Project administration; Resources; Supervision; Writing – original draft; Writing – review & editing; **Yuanyuan Li**: Conceptualization; Funding acquisition; Project administration; Resources; Supervision; Validation; Writing – original draft; Writing – review & editing

## Funding

This study was funded by the National Natural Science Foundation of China (Grant Nos. 82200581, 82200626), the General Program of China Postdoctoral Science Foundation (Grant Nos. 2025M772181, 2024M751499), General Program of Nanjing Postdoctoral Science Foundation (Grant No. 2024BHS205), and Young Scholars Fostering Fund of the First Affiliated Hospital of Nanjing Medical University (PY202412).

## Conflicts of Interest

The authors declare no conflicts of interest.

## Supporting information




**Supporting File 1**: advs76466‐sup‐0001‐Figure.docx.

**Supporting File 2**: advs76466‐sup‐Table S1‐S3.docx.

## Data Availability

The data that support the findings of this study are available from the corresponding author upon reasonable request.
