# Supplementary material for: 8‐Oxoguanine Modified CircMTUS1 Drives PABPC1 Phase Separation to Promote Gastric Cancer Progression and Cisplatin Resistance via Autophagy
Source: Adv Sci (Weinh). 2026 Jul 9;13(54):e76466. doi: 10.1002/advs.76466 (PMC13348346; doi:10.1002/advs.76466)
Supplement: Supplementary file 1 [file ADVS-13-e76466-s001.docx]

Figure S1: A-D: Heatmap of circRNA sequencing (GSE100170, GSE152309, GSE184882 and GSE248612); E: Venn plot of differentially expressed genes. F and G: qRT-PCR of has_circ_0001368(F) and has_circ_0000390(G) expression in GC tissues.

Figure S2: A: The high or low expression of circMTUS1. B-I: he associations of circMTUS1 expression with clinical stage (B), tumor depth (C), lymph node status (D), histological type (E), tumor size (F), age (G), gender (H) as determined through qRT-PCR.

Figure S3: A: qRT-PCR analysis of circMTUS1 mRNA expression after treatment with si1-circMTUS1 and si2-circMTUS1. B-D: Assessment of HGC-27 cell proliferation by EdU and colony formation assays. E: Wound healing assay to detect the effect of circMTUS1 on cell migration. F: Decreased or increased circMTUS1 regulated the migration and invasion of HGC-27 cells.

Figure S4: A and B: qRT-PCR analysis of MTUS1 mRNA expression after treatment with circMTUS1 overexpression plasmid in AGS (A) and HGC-27 (B) cell lines. C and D: qRT-PCR analysis of MTUS1 mRNA expression after treatment with MTUS1 overexpression plasmid in AGS (C) and HGC-27 (D) cell lines. E-J: Assessment of AGS and HGC-27 cells proliferation by CCK8(E, AGS; F, HGC-27), EdU(G, AGS; H, HGC-27) and colony formation assays (I, AGS; J, HGC-27). K and L: Increased MTUS1 regulated the migration of AGS (K) and HGC-27 cells (L).

Figure S5: A-C: o8G(A), m5C(B) and m6A(C) dot blot assay in GC patients. MB staining is used as a loading control. D: A: o8G dot blot assay of o8G abundance in GC cells. MB staining is used as a loading control. E: o8G expression in GC tissues tested by IHC. F: CLIP‒qPCR analysis of the o8G modification region in circMTUS1 in HGC-27 cell lines. G-J: Intracellular ROS detection and statistical analysis of ROS levels in HGC-27 cells treated with different concentrations H_2_O_2_ (G and H) and NAC (I and J). K: IF staining of o8G modification levels after treatment with H_2_O_2_ and NAC in HGC-27 cell lines. L: The RIP products were subjected to agarose gel electrophoresis after treatment with H_2_O_2_ and NAC in HGC-27 cell lines. M: RIP assay and qPCR analysis of circMTUS1 o8G modification after treatment with H_2_O_2_ and NAC. N: An RNA stabilization assay was carried out after treatment of HGC-27 cells with H_2_O_2_ and NAC.

Figure S6: A and B: The RNA level of circMTUS1 and MTUS1 in the pulldown products of circMTUS1 probe. C: circMTUS1 probes pull-down assays and silver staining for the proteins. D: MS analysis of circMTUS1 probe pulldown products. E: Bioinformatics prediction of proteins interacts with circMTUS1. F: Immunoblotting analysis of YBX1 and PABPC1 in RNA pull-down samples by circMTUS1 probes and control probes in HGC-27 cell lines. G: Co-IP followed by immunoblotting analysis of YBX1 and PABPC1 in HGC-27 cells. H: The co-localization of circMTUS1 with YBX1 in HGC-27 cell lines is detected using FISH combined with immunofluorescence assays. I: Results of circMTUS1 m5C RIP-qPCR and agarose gel electrophoresis. J: o8G RIP assay of HGC-27 cells treated with YBX1 siRNA or overexpression plasmid. K: An RNA stabilization assay was carried out after treatment of HGC-27 cells with YBX1 siRNA or overexpression plasmid. L: o8G RIP assay of AGS (upper) and HGC-27(lower) cells treated with YBX1 siRNA or overexpression plasmid. M-N: o8G-RIP qPCR analysis of YBX1 overexpression or depression after treatment with H_2_O_2_ or NAC. O and P: Further overexpress and knockdown YBX1 in HGC-27 cells treated with H2O2 and NAC, and detect the protein expression of YBX1 and PABPC1.

Figure S7: A: a RIP‒qPCR assay was performed to detect the interaction between circMTUS1 and PABPC1. B: The co-localization of circMTUS1 with PABPC1 in HGC-27 cell lines is detected using FISH combined with immunofluorescence assays. C: The impact of circMTUS1 on YBX1 and PABPC1 protein expression levels. D: IB analysis of lysates from cells transfected with NC or si-YBX1. E: IB analysis of lysates from cells transfected with vector or YBX1 overexpression plasmid. All the cells were treated with MG-132 for 8 h before harvesting.

Figure S8: A: Assessment of the proliferation of AGS and HGC-27 cells transfected with circMTUS1 and PABPC1 overexpression plasmid by a CCK-8 assay. B and C: Assessment of cell proliferation by EdU(B) and colony formation assays(C). D: the migration of AGS and HGC-27 cells.

Figure S9: A and B: Kaplan-Meier analysis of the correlation between YBX1 expression and OS (A) or OS with chemotherapy (B). C-H: Relative viability of circMTUS1 overexpression plasmid- or siRNA-transfected cells exposed to 5-FU (C and D), DOX (E and F), MMC (G and H) at the indicated concentrations for 48 h. I: Relative viability of stable circMTUS1 overexpression or knockdown AGS cells exposed to cisplatin at the indicated concentrations for 48 h. J: Cell viability of AGS and AGS/DDP (cisplatin-resistant) cells after circMTUS1/PABPC1 overexpression exposed to cisplatin at the indicated concentrations for 48 h. K-N: The circMTUS1 RNA level (K, L) and o8G-circMTUS1 level (M, N) after treated with DDP or DDP+NAC in AGS(K) and HGC-27(L) cells. O: TUNEL assays of AGS/DDP cell lines cotransfected with PABPC1 and circMTUS1 overexpression plasmids. P: The protein level of PABPC1 and YBX1 in mice tumor tissues.

Figure S10: A: Bioinformatics identifies a major IDR in PABPC1’s amino acid sequence. B: The KEGG analysis of proteins from mass spectrometry after circMTUS1 probe pull-down. C: Immunofluorescence indicating circMTUS1 (green), G3BP1(Red) and PABPC1 (Cyan) in AGS cells. The cells were incubated with SA/H2O2/DDP and transfected with circMTUS1 plasmid.

Figure S11: A and B: The circMTUS1 RNA level after treated with Rapa in AGS(A) and HGC-27(B) cells.
