# Supplementary material for: 8‐Oxoguanine Modified CircMTUS1 Drives PABPC1 Phase Separation to Promote Gastric Cancer Progression and Cisplatin Resistance via Autophagy
Source: Adv Sci (Weinh). 2026 Jul 9:e76466. Online ahead of print. doi: 10.1002/advs.76466 (PMC13348346; doi:10.1002/advs.76466)
Supplement: Supplementary file 2 — Supporting File 2: advs76466‐sup‐Table S1‐S3.docx. [file ADVS-9999-e76466-s001.docx]

Table S1: siRNA and RNA oligonucleotides sequences.

|  | Sense（5’-3’） |
| --- | --- |
| si1-circMTUS1 | CAGUUCUGGGAACAACAAATT |
| si2-circMTUS1 | GUUCUGGGAACAACAAAUCTT |
| si3-circMTUS1 | GUCCAGUUCUGGGAACAACAA |
| si4-circMTUS1 | GUGUGUCCAGUUCUGGGAACA |
| si-YBX1 | GGUUCCCACCUUACUACAU |
| si-Control | UAAGGCUAUGAAGAGAUAC |
| circMTUS1 FISH probe | T+TGTTGT+TCCCAGAACTGGACACAC |
| si-G3BP1 | ACAUUUAGAGGAGCCUGUUGCUGAA |

Table S2: The primer sequence for qRT-PCR

| circMTUS1 | F: 5’-TGATCGAGCCGTATCTCCTC-3’  R: 5’-GTGGGCAAAATGGTCTGTCT-3’ |
| --- | --- |
| GAPDH | F:5’-GCACCGTCAAGGCTGAGAAC-3’  R:5’-GGATCTCGCTCCTGGAAGATG-3’ |
| MTUS1 | F:5’-CCGGGGAGAGCTAGTCACT-3’ |
|  | R:5’-CTGCTGGACGAATGCTTCA-3’ |
| has_circ_0001368 | F:5’-ATACATTCAGCCGTCAGGTG-3’ |
|  | R:5’-TAGTTGCTGGGGAATCACGC-3’ |
| has_circ_0000390 | F:5’-CCCTGGACTGGAATGATGCT-3’ |
|  | R:5’-AGTGTTCCGAGCAGCTAGTT-3’ |
| YBX1 | F:5’-ACAAGAAGGTCATCGCAACG-3’ |
|  | R:5’-AACTGGAACACCACCAGGAC-3’ |
| PABPC1 | F:5’-CAGAGAATGGCAAGTGTACGAGC-3’ |
|  | R:5’-GCTAGGAGGATAGTATGCAGCAC-3’ |

Table S3: CLIP primer sequence

| **CLIP primer** | **Forward Sequence** |
| --- | --- |
| CLIP1-F | GACTGAAAACCTTTCAACTGTGC |
| CLIP2-F | TGAATTGCAAACCTTCTTTACCA |
| CLIP3-F | ACCCTGCTGTAGTTACTGGT |
| CLIP4-F | CTGAGAAGCCCAAATATCTGCA |
| CLIP5-F | GCTACTGTGATGCCTTGGAG |
| CLIP6-F | CCATTCTGATAAGACGCATGCA |
| CLIP7-F | ATCAGTGTGCATGTTCTTCAGG |
| CLIP8-F | GGAAGTCCCCAATGATTCTGC |
| CLIP9-F | CCCTTTAATGGTGCCAGCTT |
| CLIP10-F | AAAGGTGGGCTCGTCATTTG |
| CLIP11-F | GAGAAGGGTAATCGAGGGCT |
| CLIP12-F | AGTTACCCAAGACCAAACTTCA |
| CLIP13-F | TGAGCAGAACACCGAGATCT |
| CLIP14-F | CCAAGAACAACATCTGCCGT |
| CLIP15-F | TCCCAACATTGATAGGATTAGCC |
| CLIP16-F | TATCCAAGCCTGACTCCTGC |
